# Long-molecule scars of backup DNA repair in BRCA1- and BRCA2-deficient cancers

**DOI:** 10.1038/s41586-023-06461-2

**Published:** 2023-08-16

**Authors:** Jeremy Setton, Kevin Hadi, Zi-Ning Choo, Katherine S. Kuchin, Huasong Tian, Arnaud Da Cruz Paula, Joel Rosiene, Pier Selenica, Julie Behr, Xiaotong Yao, Aditya Deshpande, Michael Sigouros, Jyothi Manohar, Jones T. Nauseef, Juan-Miguel Mosquera, Olivier Elemento, Britta Weigelt, Nadeem Riaz, Jorge S. Reis-Filho, Simon N. Powell, Marcin Imieliński

**Affiliations:** 1 Department of Radiation Oncology, Memorial Sloan Kettering Cancer Center, New York, NY USA; 2 Department of Pathology and Laboratory Medicine, Weill Cornell Medicine, New York, NY USA; 3 New York Genome Center, New York, NY USA; 4 Physiology and Biophysics PhD program, Weill Cornell Medicine, New York, NY USA; 5 Tri-Institutional PhD Program in Computational Biology and Medicine, Weill Cornell Medicine, New York, NY USA; 6 Department of Pathology, Memorial Sloan Kettering Cancer Center, New York, NY USA; 7 Englander Institute for Precision Medicine, Weill Cornell Medicine, New York, NY USA; 8 Division of Hematology and Medical Oncology, Department of Medicine, Weill Cornell Medicine, New York, NY USA; 9 Meyer Cancer Center, Weill Cornell Medicine, New York, NY USA; 10 Department of Physiology and Biophysics, Weill Cornell Medicine, New York, NY USA; 11 Institute for Computational Biomedicine, Weill Cornell Medicine, New York, NY USA; 12 Department of Pathology and Perlmutter Cancer Center, NYU Grossman School of Medicine, New York, NY USA

**Keywords:** Homologous recombination, Breast cancer, DNA sequencing, Genome evolution, Cancer genomics

## Abstract

Homologous recombination (HR) deficiency is associated with DNA rearrangements and cytogenetic aberrations^1^. Paradoxically, the types of DNA rearrangements that are specifically associated with HR-deficient cancers only minimally affect chromosomal structure^2^. Here, to address this apparent contradiction, we combined genome-graph analysis of short-read whole-genome sequencing (WGS) profiles across thousands of tumours with deep linked-read WGS of 46 *BRCA1-* or *BRCA2*-mutant breast cancers. These data revealed a distinct class of HR-deficiency-enriched rearrangements called reciprocal pairs. Linked-read WGS showed that reciprocal pairs with identical rearrangement orientations gave rise to one of two distinct chromosomal outcomes, distinguishable only with long-molecule data. Whereas one (*cis*) outcome corresponded to the copying and pasting of a small segment to a distant site, a second (*trans*) outcome was a quasi-balanced translocation or multi-megabase inversion with substantial (10 kb) duplications at each junction. We propose an HR-independent replication-restart repair mechanism to explain the full spectrum of reciprocal pair outcomes. Linked-read WGS also identified single-strand annealing as a repair pathway that is specific to BRCA2 deficiency in human cancers. Integrating these features in a classifier improved discrimination between BRCA1- and BRCA2-deficient genomes. In conclusion, our data reveal classes of rearrangements that are specific to BRCA1 or BRCA2 deficiency as a source of cytogenetic aberrations in HR-deficient cells.

## Abstract (web summary)

Linked-read whole-genome sequencing reveals patterns of structural DNA variants that are specific to homologous recombination deficiency and can be used to distinguish between BRCA1- and BRCA2-deficient phenotypes.

## Main

Cancer genomes provide a record of the genetic alterations acquired from DNA damage and DNA repair defects during normal cell development and carcinogenesis^3^. Genome-wide somatic alteration patterns in BRCA1-deficient (BRCA1d) and BRCA2-deficient (BRCA2d) cancers^2,4^ are attributed to a deficiency in HR, a major pathway for the repair of double-strand breaks (DSBs) in human cells. Some of these mutational patterns could reflect specific error-prone mechanisms of DSB repair that cells use in the absence of HR^5^. Such mutational patterns can provide biomarkers of HR deficiency and help to identify clinically relevant therapeutic vulnerabilities^6,7^.

Impaired DSB repair in HR-deficient (HRD) cells is thought to compromise structural genomic integrity, leading to characteristic cytogenetic alterations including radial chromosomes and chromosome bridges^1,8,9^. Confirming these cytogenetic observations, microarray and WGS studies have found loss of heterozygosity (LOH) and other megabase-scale patterns of allelic imbalance to be enriched among HRD cancers^8,10–13^. Such copy number alterations, however, are also found in HR-proficient (HRP) tumours and have not been linked to specific classes of structural variants (SVs). Paradoxically, the key genomic features that distinguish BRCA1d and BRCA2d from HRP tumours are single-nucleotide variants (SNVs), small deletions with microhomology, tandem duplications and simple deletions^2^, all which have minimal effects on chromosomal structure. As a result, it is still poorly understood how aberrant DSB repair produces the associated cytogenetic phenotype in HR deficiency.

Developments in the analysis of cancer genomes allow for systematic annotation of complex SVs such as chromothripsis (chromosome shattering)^14^, chromoplexy (balanced rearrangement chains)^15^ and templated insertion chains (TICs)^16–18^. Previous WGS analyses of HR deficiency, however, have not considered this expanded SV taxonomy, either ignoring complex SVs^4^ or treating them as a single ‘clustered rearrangement’ category^2^. They have also treated copy number and rearrangement independently, unlike more recently developed genome-graph algorithms that integrate these features under the principle of mass balance^18^. We reasoned that a genome-graph analysis might uncover HR-deficiency-specific patterns of complex SVs, which could improve the classification of HRD tumours and provide mechanistic insights into their origin.

## Genome-graph analysis of HRD tumours

**Extended Data Fig. 1:**
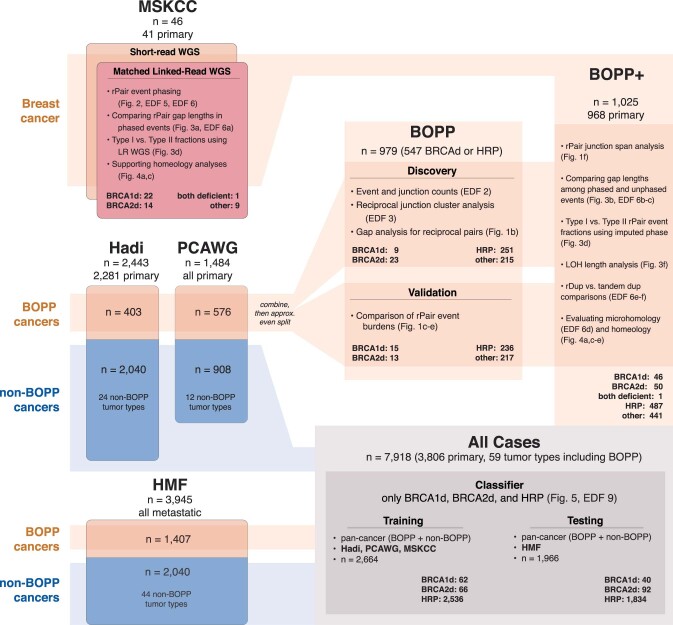
Overview of datasets and analyses. Schematic illustrating cases included for analysis in the present manuscript. MSK, Memorial Sloan Kettering Cancer Center. PCAWG, Pan-Cancer Analysis of Whole Genomes/International Cancer Genome Consortium. HMF, Hartwig Medical Foundation. BOPP, breast, ovarian, pancreas, and prostate. Hadi refers to WGS profiles from a previously published pan-cancer analysis (ref. ^18^). Cohorts are mapped to key analyses in the study, denoted by main and Extended Data figure (EDF) panels.

To investigate the role of complex SVs in HRD cancers, we assembled a dataset of 979 predominantly primary (95%) cancer WGS profiles from four tumour types that are commonly associated with HR deficiency^19^ (breast, ovarian, prostate and pancreatic cancer; referred to as BOPP, Methods and Extended Data Fig. 1). These included 24 and 36 cancers with biallelic inactivation of *BRCA1* and *BRCA2*, respectively, and 487 HRP tumours that lacked mono- or biallelic alterations in any HR-pathway gene (Extended Data Fig. 1, Supplementary Table 1, Methods and Supplementary Note 1). We then compared SV patterns between BRCA1d, BRCA2d and HRP tumours using methods that integrate copy number changes and rearrangements across genome graphs^18^.

**Extended Data Fig. 2:**
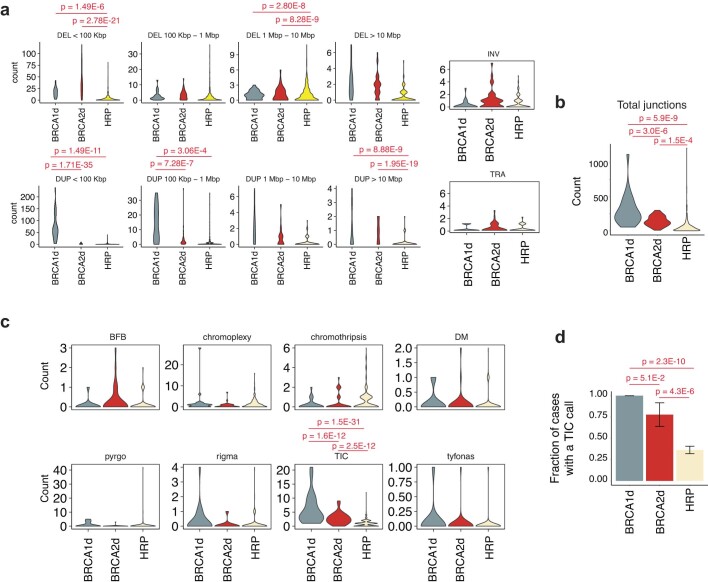
Comparing the burden of SV classes across genotypes. **a**–**c**, Comparison of the burden (count) of simple SV classes (**a**), total junctions (**b**) and complex SV classes (**c**) across BRCA1d (*n* = 9), BRCA2d (*n* = 23) and HRP cases (HRP, n = 251). Templated insertion chains (TICs): BRCA1d vs HRP, RR 6.56, P = 1.5 x 10^−31^, BRCA2d vs HRP, RR 2.96, P = 2.5 x 10^−12^. P values obtained by two-sided Wald test on a gamma-Poisson regression model. (BFB, breakage–fusion–bridge. DM, double minute. TIC, templated insertion chain.) **d**, Fraction of cases with at least one TIC event across BRCA1d (*n* = 9 tumours with TIC/*n* = 9 tumours), BRCA2d (*n* = 17 tumours with TIC/*n* = 23 tumours) and HRP cases (*n* = 90 samples with TIC/*n* = 251 tumours). Error bars show 95% confidence interval on the Bernoulli trial parameter. *P* values and odds ratios obtained by Fisher’s exact test. Source data

Analysing the burden of individual simple SV classes between BRCA1d, BRCA2d and HRP tumours, we confirmed the previously observed enrichment of short (1–100 kbp) SV duplications in BRCA1d cancers, and deletions in both BRCA1d and BRCA2d cancers (Extended Data Fig. 2a). Although BRCA1d and BRCA2d cancers had higher SV burdens than did HRP cancers (Extended Data Fig. 2b), we found no significant difference in the burden of simple translocations and inversions (Extended Data Fig. 2a), as has been previously noted^4^.

We next asked whether HRD tumours were enriched in specific classes of complex SVs, and found no significant difference in the burden of seven previously characterized complex SV categories^18^ in BRCA1d or BRCA2d relative to HRP tumour samples (Extended Data Fig. 2c). Contrary to the commonly held assumption that HRD cancers are exceptionally rearranged compared to HRP cancers, we found that they contained similar burdens of most SV classes, including complex SVs. TICs, however, were significantly enriched among both BRCA1d and BRCA2d relative to HRP tumours (Extended Data Fig. 2c). TICs arise through the copying and pasting of smaller (less than 10 kb) and genomically dispersed DNA segments in between larger (megabase-scale) segments^16^.

## Near-reciprocal SVs in HRD cancers

**Fig. 1:**
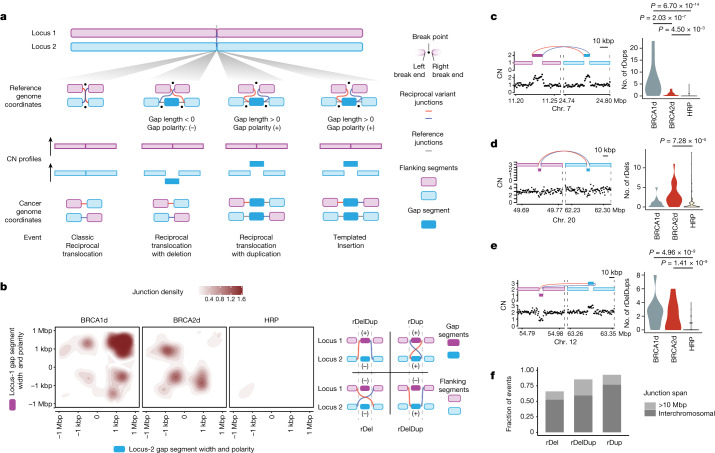
Reciprocal pairs are enriched in BRCA1d and BRCA2d tumours. **a**, Schematic of exact and near reciprocality, using translocations as an example. Exactly reciprocal junctions link break ends that adjoin the same break point (schematic on the right), giving rise to a balanced translocation. Near-reciprocal junctions are associated with a gap segment (dark blue) that is lost (middle left) or gained (right examples). The gap segment polarity refers to whether the adjoining junctions connect to the gap segment (+ polarity; right two examples) or to its adjacent segments (− polarity, middle left example). The polarity determines whether there is a copy gain (+) or loss (− polarity) of the gap segment. Both (−) and (+) gap segments can give rise to quasi-balanced translocations; however, (+) gap segments are also equally consistent with a templated insertion (right). CN, copy number. **b**, Gap segment lengths and polarities of three canonical reciprocal pair patterns (right) plotted across BRCA1d, BRCA2d or HRP cases (left). Density is calculated as a Gaussian kernel normalized by the number of BRCA1d (*n* = 9), BRCA2d (*n* = 23) or HRP (*n* = 251) cases in each plot. **c**–**e**, Examples of rDups (**c**), rDels (**d**) and rDelDups (**e**) with violin plots showing their relative burdens across 15 BRCA1d, 13 BRCA2d and 236 HRP samples, which are independent from the data in **b**. *P* values obtained by Wald test on a gamma-Poisson regression model. **f**, Distribution of junction spans associated with different classes of reciprocal pair SVs. Note that junction span is distinct from gap segment length; the former refers to the genomic distance between the two break ends belonging to a junction, whereas the latter refers to the distance between reciprocal break ends belonging to distinct junctions. Source data

A classic reciprocal rearrangement (that is, balanced translocation or inversion) occurs without the loss or gain of genetic material and involves a pair of DNA junctions with break ends that adjoin the same break point (Fig. 1a, left). However, many rearrangements, including translocations and inversions, are near-reciprocal, with break ends that are nearby but not adjacent on the genome (Fig. 1a, middle). TICs^16^ and chromoplexies^15^ (chains of balanced rearrangements) are examples of complex SVs that are near-reciprocal.

Near-reciprocal SVs contain copy loss or gain of the intervening genomic region, which we call a gap segment (Fig. 1a, middle). The direction of copy loss versus gain at the gap segment is determined by its polarity, which by conservation of mass yields a copy gain when break ends join the gap segment (+ polarity) and copy loss when break ends join the flanking segments (− polarity). For a (+) gap segment, the identical locus topology and copy number profile may be equally consistent with a translocation or a simple templated insertion, in which a gap segment is copied to a distant locus and leaves the source locus unaltered (Fig. 1a, right).

**Extended Data Fig. 3:**
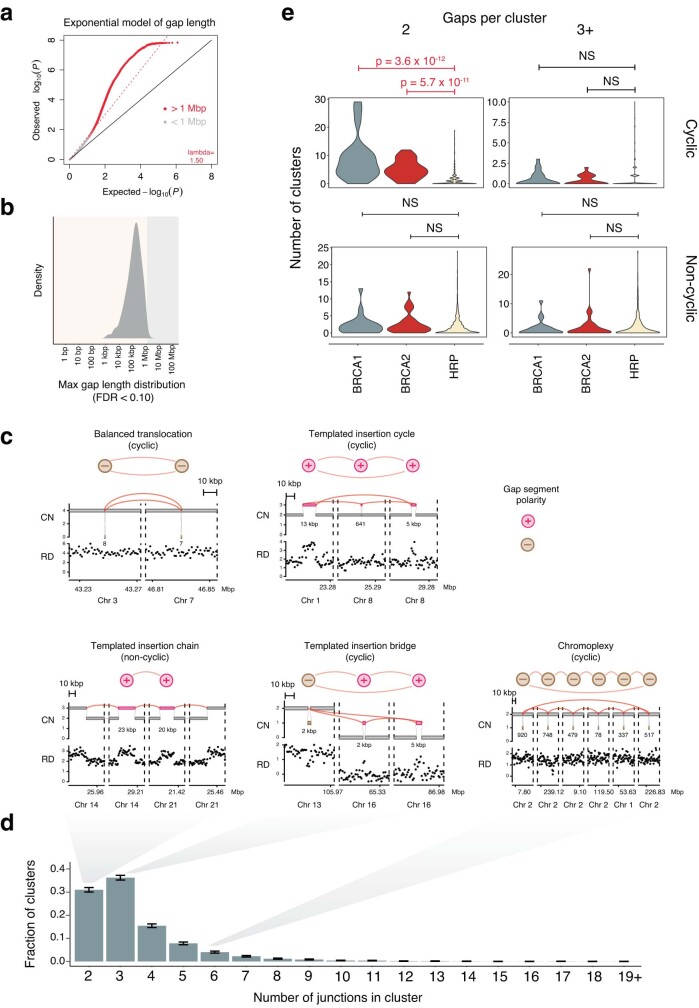
Characteristics of reciprocal junction clusters. **a**, Quantile-quantile plot of observed −log_10_ *P* values obtained by evaluating 473,382 observed gap segment lengths from 283 tumour samples against an exponential null model (see Methods). The *x* axis represents -log_10_ transformed quantiles from the uniform distribution. Gap segment lengths less than 1 Mbp are shown in red. **b**, Density plot showing distribution of gap segment lengths with FDR < 0.1 across 283 tumour samples the vast majority of which are less than 1 Mbp. **c**, Examples of 2-, 3- and multi-way (based on gap segment number) reciprocal SV topologies, including a spectrum of simple and complex SV classes. **d**, Histogram of reciprocal SV cluster lengths across 283 tumour samples and 1,854 junctions. **e**, Violin plots comparing cyclic versus linear and 2-way versus higher order (3+ way) reciprocal SV topologies across 283 tumour samples. Error bars show 95% confidence interval on the Bernoulli trial parameter. *P* values obtained by two-sided Wald test on a gamma-Poisson regression model. Source data

Despite their enrichment in BRCA1d and BRCA2d cancers, TICs were still found in a substantial fraction (36%) of HRP tumours (Extended Data Fig. 2d). We hypothesized that a more comprehensive analysis of near-reciprocal junctions might yield uniquely HR-deficiency-specific patterns. Analysing clusters of near-reciprocal junctions linked by (+) and (−) polarity gaps (Methods and Extended Data Fig. 3a,b) in a training dataset (Extended Data Fig. 1) revealed simple paired patterns (for example, balanced translocations with short 6–7-bp (−) polarity gap segments), as well as more complex cyclic and non-cyclic reciprocal SV topologies comprising (+) and/or (−) polarity gap segments (Extended Data Fig. 3c,d).

Comparing near-reciprocal SV topologies across genotypes, we found that paired and cyclic patterns were most significantly enriched in BRCA1d (*μ* = 10.17 events per case, relative risk (RR) 4.72, *P* = 3.6 × 10^−12^, Wald test on gamma-Poisson regression) and BRCA2d (*μ* = 5.59, RR 4.73, P = 5.7 x 10^−11^) cancers relative to HRP cancers (*μ* = 1.13) (Extended Data Fig. 3e). We call these cyclic and paired patterns reciprocal pairs. Notably, we did not find genotype-specific differences in non-cyclic or higher-order reciprocal SVs comprising more complex templated insertion events and chromoplexies (Extended Data Fig. 3c–e).

Visualizing the gap segment lengths and polarities of reciprocal pairs revealed three distinct subpatterns that were specific to BRCA1 deficiency and/or BRCA2 deficiency (Fig. 1b). The first was an enrichment in BRCA1d tumours of reciprocal pairs with two 100-bp–100-kbp (+) polarity gap segments, which we call reciprocal duplications (rDups; Fig. 1c, left). The second was an enrichment in BRCA2d tumours of reciprocal pairs with two 1-bp–10-kbp (−) gap segments, which we call reciprocal deletions (rDels; Fig. 1d, left). The third was an enrichment in BRCA1d and BRCA2d cases of reciprocal pairs comprising 1-bp–100-kbp gap segments of opposite (+) and (−) polarity, which we call reciprocal deletion-duplications (rDelDups; Fig. 1e, left). Inspection of individual reciprocal pair loci (Fig. 1c–e, left) confirmed that these occurred in genomic regions that otherwise did not contain other rearrangements within a 1-Mbp vicinity. Analysis of these patterns in a validation dataset (Extended Data Fig. 1) confirmed the enrichment of rDups, rDels, and rDelDups in BRCA1, BRCA2 and HR deficiency, respectively (Fig. 1c–e, right and Supplementary Note 2).

## Long-molecule WGS of HRD cancers

**Extended Data Fig. 4:**
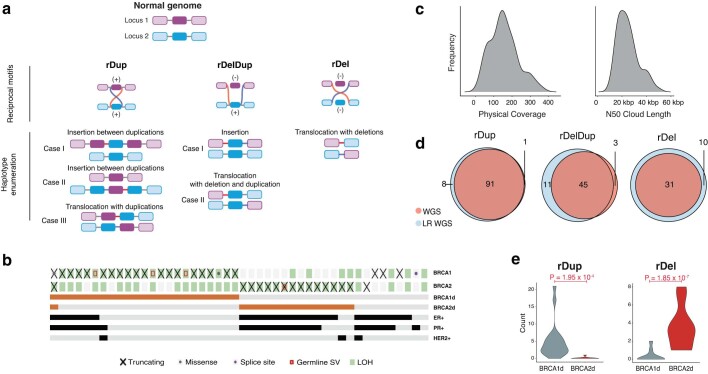
Haplotype deconvolution of reciprocal pairs with LR WGS validates reciprocal pairs in *BRCA1-* and *BRCA2* mutated cancers. **a**, Enumeration of all possible phased allelic deconvolutions of each of the three reciprocal-pair classes (rDups, rDelDups and rDels). **b**, Molecular and pathological characteristics of the 46 breast cancer cases profiled with LR WGS in the MSKCC cohort. **c**, Sequencing library characteristics across 92 LR WGS profiled samples. **d**, Concordance between LR WGS and short-read WGS reciprocal-pair calls among BRCA1d (*n* = 22) and BRCA2d (*n* = 14) cases. **e**, Comparison of rDup and rDel counts among BRCA1d (*n* = 22) and BRCA2d (*n* = 14) cases within the MSKCC cohort. *P* values obtained by two-sided Wald test on a gamma-Poisson regression model. Source data

Identical rearrangement topologies can have very distinct chromosomal outcomes, or phases (Fig. 1a). We noted that the topology and copy number profile of reciprocal pairs were consistent with either of two outcomes: (1) the templated insertion of a small (around 1 bp–100 kbp) segment to an ectopic site; or (2) a larger quasi-balanced rearrangement such as a translocation or inversion (Extended Data Fig. 4a). We also observed that most reciprocal pairs comprised long-range (either interchromosomal or larger than 10-Mbp intrachromosomal) junctions (Fig. 1f). This indicated that, depending on the outcome, reciprocal pairs could have either a minimal or a major effect on chromosomal structure.

To resolve this aspect of reciprocal pairs, we performed linked-read (LR) and standard WGS on 46 tumours and matched normal samples that were originally found by clinical panel sequencing to have inherited or somatic mutations in *BRCA1* (27 cases) or *BRCA2* (19 cases; Extended Data Figs. 1 and 4b and Supplementary Note 3). LR WGS provided deep (median, 149×) genome-wide physical coverage of tumour and normal samples through barcoded short-read sequencing of long DNA molecules (median length, 24.4 kbp; Extended Data Fig. 4c). We reasoned that long molecules would help to resolve reciprocal pairs into phased somatic haplotypes and provide insight into the mechanistic origin and outcome of these SVs.

We identified 186 reciprocal pairs among BRCA1d and BRCA2d cases (*μ* = 5.17 per case). Comparison of standard and LR WGS profiles showed concordant rDel, rDup and rDelDup calls (83.5% overlap; Extended Data Fig. 4d), although LR WGS identified 29 additional reciprocal pairs. Confirming results from the BOPP short-read WGS dataset, BRCA1d tumours had higher burdens of rDups than did BRCA2d tumours (*P* = 1.95 × 10^−4^, RR = 49.50, Wald test on gamma-Poisson regression), and rDups were found in most (82%) BRCA1d tumours but in only one BRCA2d tumour. Similarly, rDels were present in most (71%) BRCA2d tumours but absent in all but four BRCA1d tumours (*P* = 1.85 × 10^−7^, RR = 11.67; Extended Data Fig. 4e).

**Fig. 2:**
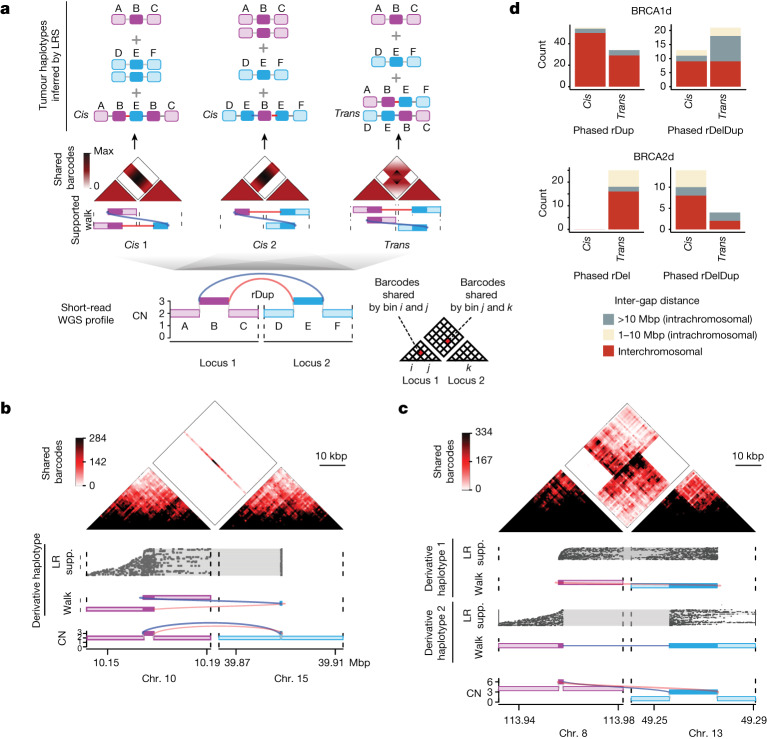
LR WGS reveals *cis* and *trans* phases for similar reciprocal pair topologies. **a**, Multiple phased allelic reconstructions are consistent with the rDup SV pattern observed in short-read WGS. Each derivative allele is represented as a ‘walk’ or oriented sequence of reference genomic segments. A phased reconstruction comprises a set of derivative alleles that together account for junction and segmental copy numbers observed in the short-read WGS genome graph. For a rDup, two of the three possible reconstructions (*cis* 1 and *cis* 2) place junctions adjacently, corresponding to the templated insertion of a distant segment between duplicated copies of a gap segment at the source locus. In the third case (*trans*), the junctions are located on discontiguous or distant alleles, consistent with a large translocation or inversion, respectively, in which each derivative allele contains a copy of both gap segments. Each reconstruction has a distinct LR WGS footprint, as visualized by a heat map in which the pixels represent LR WGS barcode sharing between rearranged loci (bottom right schematic, also applicable to **b** and **c**). LRS, linked-read sequencing. **b**,**c**, Two rDups, each phased by LR WGS, in *cis* (**b**) and in *trans* (**c**). **d**, Counts of LR WGS phased rDups, rDels and rDelDups from either BRCA1d (top; *n* = 22) or BRCA2d (bottom; *n* = 14) tumours. Reciprocal pairs are coloured by their junction span (1–10 Mbp, >10 Mbp or interchromosomal), see Fig. 1f for explanation. Source data

**Extended Data Fig. 5:**
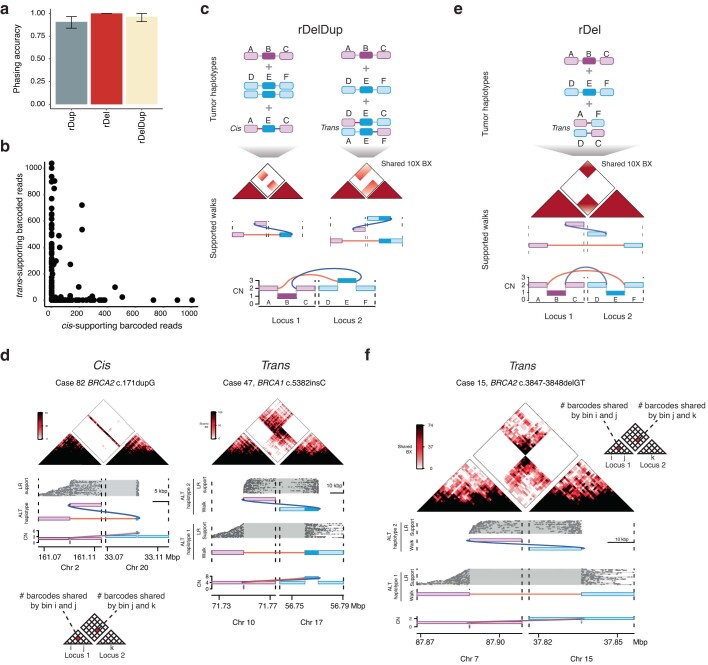
Phasing rDels and rDelDups with LR WGS. **a**, Accuracy of phasing algorithm across simulated LR data sampled from *cis* or *trans* allelic configurations of 268 observed rDel, rDelDup and rDel loci. Number of correct calls/number of total events for each rPair event are as follows: rDup: 108/160, rDelDup: 61/68, rDel: 39/40. Error bars show 95% confidence interval on the Bernoulli trial parameter. **b**, Number of LR barcodes supporting *cis* versus *trans* configuration for each of the 186 reciprocal pair loci observed across 46 tumours profiled with LR WGS. **c**,**e**, Predicted LR profiles for an rDelDup (**c**) or rDel (**e**). Each plot shows a genome graph (track labelled ‘CN’, copy number) and possible allelic walks in reference coordinates (track labelled ‘Supported walks’) together with the barcode sharing heat map (middle top) and resulting *cis* and/or *trans* haplotypes in the cancer genome. In each plot, the track labelled ‘Supported walks’ represented enumerated paths in reference coordinates through the corresponding graph (‘CN’ track) with LR data supporting the barcode sharing heat map above (‘LR support’ track). Each heat map shows the number of barcodes shared by pairs of bins within and between locus 1 and 2. The ‘Tumour haplotypes’ track shows the total phased linear alleles in somatic coordinates inferred based on the LRS data. **d**,**f**, Examples of a (**d**) *cis* and *trans* rDelDup and (**f**) *trans *rDel across three BRCA1d or BRCA2d cases. CN, copy number. ALT, rearranged haplotype. BX, linked-read barcodes. LRS, linked-read WGS. Source data

To assess whether reciprocal pairs could be responsible for large-scale rearrangements in HRD cancers, we inferred their derivative chromosomal structure, or phase (Extended Data Fig. 4a). The specific goal of these analyses was to distinguish between *cis* (copy-paste, templated insertion) outcomes and *trans* (balanced translocation or inversion) outcomes on the basis of LR WGS alignment patterns (Fig. 2a and Extended Data Fig. 4a; Methods). After benchmarking phasing methods (Extended Data Fig. 5a,b and Supplementary Note 4), we analysed reciprocal pairs in our LR WGS data.

Phasing of 94 rDups in BRCA1d samples revealed a predominance (67/94; 71%) of *cis* phases (Fig. 2b,d), each resulting in the copying and pasting of a gap segment in between the tandem-duplicated gap segments of a distant locus. For example, given two loci ABC and DEF, this would yield an ABEBC haplotype containing the variant BE and EB junctions in tandem, and leaving the other DEF haplotype unrearranged (Fig. 2b). The remaining 29% of loci contained *trans* configurations (Fig. 2c,d), in which the BE and EB junctions were placed on discontiguous (for interchromosomal rDups) or distant (for intrachromosomal rDups) rearranged alleles. In these outcomes, two distinct derivative alleles ABEF and DEBC shared the duplicated B and E segments. This included balanced translocations with up to around 20 kbp of duplicated sequence at the junction (Fig. 2c).

Similarly, we used LR WGS to phase 46 rDelDups across 37 HRD cases (22 BRCA1d, 14 BRCA2d and one both BRCA1d and BRCA2d). As with rDups, we found both *cis* and *trans* phases at various loci (Extended Data Fig. 5c,d), although with a *trans* predominance of around 2:1 in BRCA1d tumours and a *cis* predominance of around 4:1 in BRCA2d tumours (Fig. 2d). Given ABC and DEF loci, *cis* rDelDups comprised a ‘cut, copy and paste’ outcome with an additional copy of E replacing B on a derivative AEC allele, with an unrearranged DEF locus containing the other E copy; by contrast, *trans* loci showed the same (1–241 kbp) E segment duplicated across two distinct DEC and AEF derivative loci. Finally, BRCA2d-tumour*-*specific rDels were predicted by short reads to give rise to strictly *trans* outcomes, which was confirmed by LR WGS (Fig. 2d and Extended Data Fig. 5e,f). These results show that *trans* reciprocal pairs are frequent among rDups, rDels and rDelDups and serve as a source of large-scale rearrangements in BRCA1d and BRCA2d tumours.

## Chromosomal effect of *trans* reciprocal pairs

**Fig. 3:**
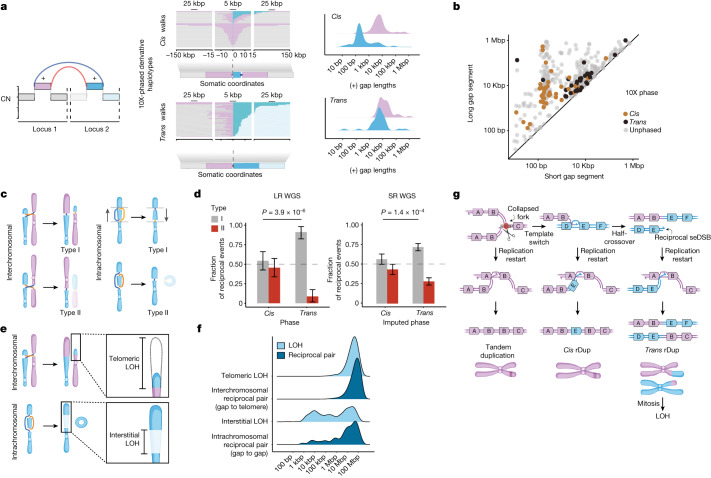
Aberrant replication-restart model links *trans* reciprocal pairs to megabase-scale chromosomal alterations. **a**, Derivative haplotypes (middle) for *cis* and *trans* rDups from BRCA1d (*n* = 22) and BRCA2d (*n* = 14) LR WGS profiles. Somatic chromosomal coordinates were harmonized so that 0 corresponds to the location of the first junction in the walk, with intervals coloured according to the genome-graph schematic (left). Density (right) shows the distribution of (+) gap segment lengths within *cis* and *trans* phased derivative chromosomes resulting from rDups. **b**, Scatter plot of longer and shorter (+) gap segment lengths for each rDup across BRCA1d (*n* = 46) and BRCA2d (*n* = 50) BOPP cases coloured according to LR WGS phase. **c**, Schematic illustrating two chromosomal outcomes of *trans* reciprocal pairs that either maintain centromere dosage (type I) or create an acentric derivative (type II). **d**, Fraction of type I and type II orientations among observed (left; LR WGS, *n* = 131 events) and imputed (right; short-read (SR) WGS, *n* = 593 events) *cis* and *trans* reciprocal pairs. *P* values obtained by two-sided Fisher’s exact test. Error bars: 95% confidence interval on Bernoulli trial parameter. **e**, Schematic of LOH outcomes after a *trans* reciprocal pair. **f**, LOH length distributions plotted versus gap-to-telomere and gap-to-gap lengths for inter- and intrachromosomal reciprocal pairs, respectively, among BRCA1d (*n* = 46) and BRCA2d (*n* = 50) BOPP cases. **g**, Proposed replication-restart model linking tandem duplications, *cis* and *trans* rDups and LOH. Locus 1 (ABC) undergoes replication-fork collapse and may invade locus 2 (DEF), giving rise to *cis* and *trans* rDups. The latter might lead to LOH (after mis-segregation). seDSB, single-ended DSB. Variations of this model for rDelDups and rDels are shown in Extended Data Fig. 6g,h. Diagrams in **c**, **e**, **g** created with BioRender.com. Source data

Given the distinct chromosomal outcomes of *cis* versus *trans* reciprocal pairs, we looked for features that could distinguish these loci in short-read WGS and thus enable the study of reciprocal pair phase across a larger dataset. Deeper analysis and visualization of the phased structure of *cis* rDups (Fig. 3a, top) revealed that a short (50 bp–1 kbp) E segment was predominantly interleaved between two copies of a long (1–300 kbp) B segment in an ABEBC configuration. The trans rDups, however, comprised pairs of longer (1 kbp–300 kbp) B and E segments in ABEF and DEBC haplotypes (Fig. 3a, bottom).

**Extended Data Fig. 6:**
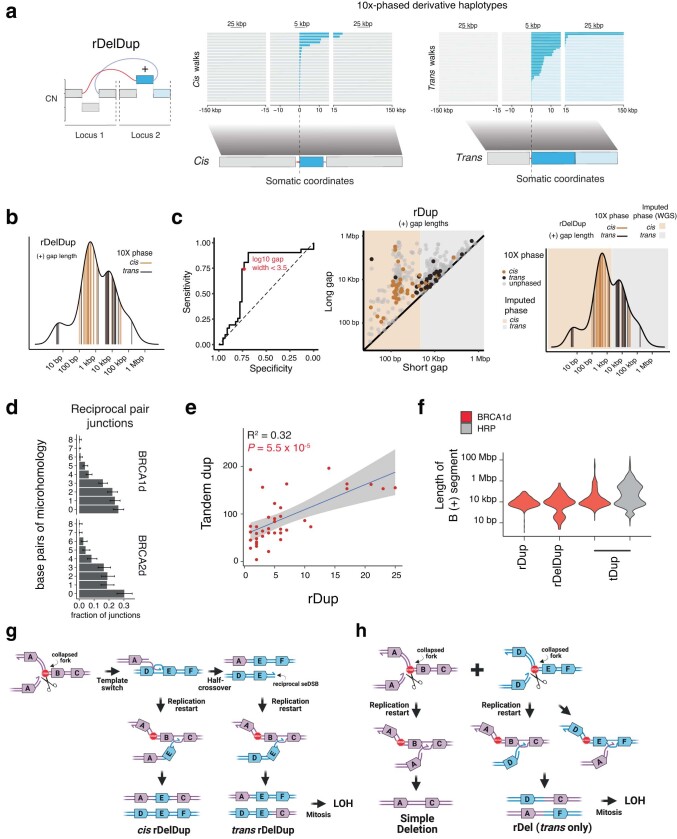
Links between reciprocal pairs, LOH and tandem duplications. **a**, Schematic defining the (+) gap segment of rDelDup reciprocal pairs (left). The (+) gap segment lengths of *cis* and *trans* phased haplotypes from BRCA1d (*n* = 22) and BRCA2d (*n* = 14) samples are shown in the centre and right panels, respectively. **b**, (+) gap segment length distribution of unphased rDelDup reciprocal pairs across *n* = 96 BRCA1d or BRCA2d WGS samples with vertical lines denoting lengths of LR WGS phased events across *n* = 36 BRCA1d or BRCA2d samples. **c**, ROC curve for *cis*/*trans* phasing using gap segment length threshold (left panel) with selected value shown in red (*cis* phase is imputed if the log_10_ length < 3.5). rDup (centre) and rDelDup (right) gap segment length distributions with background colour showing the length threshold used for phase imputation. **d**, Base pairs of microhomology at 644 and 328 reciprocal-pair junctions across 46 BRCA1d and 50 BRCA2d tumours, respectively. Error bars show 95% confidence interval on the Bernoulli trial parameter. **e**, rDup and tandem duplication count per sample across 46 BRCA1d samples. *R*^2^ and *P* value obtained by two-tailed Spearman rank correlation. **f**, Violin plots showing length of the longer rDup gap segment, (+) rDelDup gap segment and tandem duplication segment in BRCA1d (red) (*n* = 46) and HRP (*n* = 487) samples (grey). *P* values obtained by two-tailed Wilcoxon rank-sum test. **g**,**h**, Extensions of the aberrant replication-restart model for rDups (Fig. 3g) can be used to explain rDelDups (**g**) and rDels (**h**) around a locus (ABC) that undergoes replication-fork collapse and invades a second locus (DEF), resulting in distinct phased outcomes including *trans* configurations that can lead to LOH in subsequent cell cycles. Diagrams in **(g)** and **(h)** created with BioRender.com. Source data

Plotting these LR WGS phased *cis* and *trans* loci alongside unphased data from the short-read WGS BOPP dataset, oriented to the length of the longer gap segment in each pair (*y* axis), revealed two distinct rDup clusters. The first ‘long–short’ rDup cluster involved the linking of longer (1–100 kbp) and shorter (10 bp–1 kbp) gap segments and comprised exclusively *cis* events. By contrast, *trans* rDups were entirely contained in the second ‘long–long’ cluster (Fig. 3b). Similar length differences were found to differentiate *cis* and *trans* rDelDups (Extended Data Fig. 6a,b and Supplementary Note 5). These length differences made it possible to impute reciprocal pair phase in short-read WGS with reasonable accuracy (Extended Data Fig. 6c and Supplementary Note 6; Methods).

Because *trans* reciprocal pairs might engender long-range SVs (for example, balanced translocations and inversions), we predicted that *trans* but not *cis* events would be constrained in their chromosomal orientation. Specifically, *trans* reciprocal pairs can occur in one of two centromeric orientations: the first (type I) orientation generates only monocentric chromosomes, whereas the second (type II) generates one or more acentric derivatives (Fig. 3c). As acentric DNA fragments are prone to loss in subsequent cell divisions, a junction residing on an acentric fragment will be preferentially lost and the remaining junction will not be detected as a reciprocal pair. This will result in a type I bias for *trans* reciprocal pairs. Conversely, because templated insertions do not alter the chromosomal dosage of centromeres, *cis* loci should be agnostic to type I versus type II orientation.

Indeed, when we analysed our LR WGS phased data, we found that *trans* loci had a bias of more than 9:1 towards type I versus type II, whereas *cis* loci were equally likely to be in either the type I or the type II orientation (*P* = 3.9 × 10^−6^, odds ratio (OR) = 8.57, Fisher’s exact test; Fig. 3d, left). We next analysed unphased reciprocal pairs in the short-read WGS BOPP dataset, imputing *cis* and *trans* phase on the basis of size and orientation (Fig. 3a,b and Extended Data Fig. 6a–c; Methods). We found the same direction of bias in these data (*P* = 1.4 × 10^−4^, OR = 1.96; Fig. 3d, right), despite having only imputed phases. In particular, these analyses showed that intrachromosomal *trans* reciprocal pairs yield megabase-scale inversions similarly constrained by centromere dosage.

We also found that the size distributions of interstitial and telomeric losses predicted to occur with *trans* reciprocal pairs mirrored the distribution of interstitial and telomeric LOH found in HRD cancers (Fig. 3e,f and Supplementary Note 7). Together, these results suggest that *trans* reciprocal pairs have large-scale chromosomal consequences, and thus can be implicated in cytogenetically visible aberrations that are classically associated with *BRCA1* and *BRCA2* inactivation.

## Replication-restart model of reciprocal pairs

The observation of distinct *cis* and *trans* phases arising from nearly identical junction topologies (for example, rDups) suggested that they could represent distinct outcomes of a shared DNA-repair intermediate. Notably, most reciprocal pairs joined distant genomic locations (Figs. 1f and 2d) and had minimal sequence homology (Extended Data Fig. 6d), suggesting a possible homology-independent repair mechanism. The aberrant restart of a broken replication fork^20^ has been implicated in the genesis of around 10–100-kbp tandem duplications in BRCA1d tumours^21^. Indeed, a key role of HR in human cells is in the repair of single-ended DSBs, which can arise at stalled replication forks^22–25^.

To investigate the possibility of a shared mechanism between tandem duplications and reciprocal pairs, we analysed their distributions in our data. Notably, tandem duplications, rDups and rDelDups frequently co-occurred in BRCA1d cases (Extended Data Fig. 6e). In addition, the size distribution of the longer (+) gap segments in rDups or rDelDups, but not the shorter (+) gap segments in rDups, closely mirrored that of BRCA1d-tumour-specific tandem duplications (Extended Data Fig. 6f). Although the underlying mechanism will require experimental confirmation, we found that several simple extensions to the replication-restart model could explain the full spectrum of reciprocal pair alterations, including rDups (Fig. 3g), rDelDups (Extended Data Fig. 6g) and rDels (Extended Data Fig. 6h).

Replication restart (for example, break-induced replication; BIR) mechanisms are known to be prone to template switching and half-crossovers, and have previously been linked to templated insertions in cancer cells^23,26–28^. A key decision point between template switching and half-crossover in BIR rests on the fate of the displacement loop after strand invasion^23,29^. Factors that promote displacement-loop disassembly or nascent strand displacement favour template switching with shorter replication tracts^30^. By contrast, factors that stabilize the displacement loop favour long-tract synthesis and half-crossover formation^30^. In our data, rDups and rDelDups with short ectopic (E segment) tracts were exclusively in *cis*, consistent with a template switch (Fig. 3a–d). In addition, for *cis* rDups associated with BRCA1 deficiency, the shorter E segment was always copied between two longer B segments (Fig. 3a). By contrast, only half-crossover outcomes (*trans* rDups and rDelDups) were observed alongside longer E (1–100 kbp) segments (Fig. 3a,b). In both outcomes, the distribution of rDup B-segment lengths mirrored the size distribution of tandem duplications in BRCA1d tumours (Fig. 3a and Extended Data Fig. 6f).

Together, our observations suggest that reciprocal SVs can be found in both *cis* and *trans* forms, with topological and tract-length characteristics previously associated with half-crossover and templated insertion outcomes of BIR^29,30^. We propose a provisional model invoking microhomology-mediated BIR (Fig. 3g and Extended Data Fig. 6g–h), extended from experimentally validated models established for BRCA1d-associated tandem duplications^20^, that plausibly accounts for the full spectrum of reciprocal pairs.

## Scars of backup repair in BRCA2d tumours

Given our LR WGS data, we posited that other scars of HR-deficiency-specific repair pathways could be detected using long-molecule mapping. Single-strand annealing (SSA) is a DSB repair pathway that involves the hybridization of approximately homologous (homeologous) repeat sequences flanking a DSB. Experimental model systems of HR deficiency have shown that SSA is active in BRCA2d but not in BRCA1d cancers^31–33^. SSA can tolerate as little as 80% sequence identity when annealing similar sequences deep inside resected break ends^33^; however, previous genome studies of HR deficiency have only analysed exact microhomology and have not examined inexact sequence identity.

**Fig. 4:**
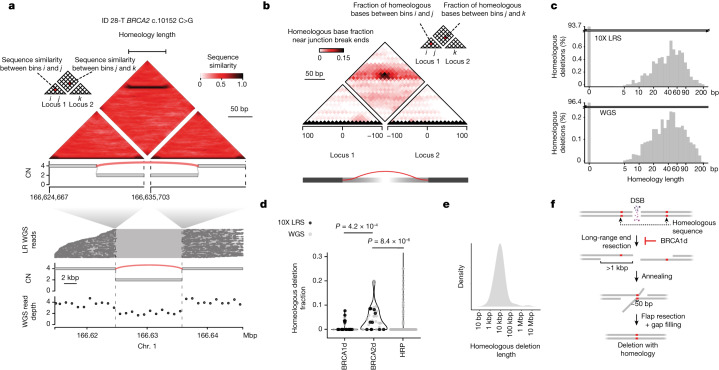
LR WGS uncovers footprints of SSA in BRCA2d genomes. **a**, Example of homeology (inexact sequence homology) across a deletion junction in a BRCA2d case detected by LR WGS. Heat map (top track) shows reference sequence similarity across pairs of 41-bp bins flanking junction break ends (Methods). Homeologous bin pairs are those with higher than 80% sequence similarity. The black line represents a continuous run of homeology, inferred through image analysis (Methods). Bottom track shows barcoded LR WGS read alignments supporting the homeologous deletion junction, in which each *y*-axis position represents a distinct LR barcode. **b**, Heat map showing counts of bases with homeology (sequence similarity ≥ 0.8) across all detected LR and standard WGS junctions (*n* = 1,240 junctions) across BRCA1d (*n* = 125), BRCA2d (*n* = 198) and HRP (*n* = 917) on a coordinate system defined around the location and orientation of each junction break end. Pixels are coloured according to the sequence similarity of the corresponding bin pair. **c**, Length of homeology (Methods) measured across all LR (top) (*n* = 34 samples, 724 junctions) and WGS (bottom) (*n* = 60 samples, 2,101 junctions) deletions. **d**, Fraction of homeologous junctions among simple deletions (more than 1 kbp) among BRCA1d (*n* = 43), BRCA2d (*n* = 46) and HRP (*n* = 374) tumours. *P* values obtained by two-sided Wald’s test on gamma-Poisson regression. **e**, Junction span (distance between break ends) of homeologous LR WGS and short read WGS deletions in BRCA2d tumours (*n* = 46). **f**. Schematic of SSA mechanism, including dependence on long-range end resection and BRCA1 function. Diagram created with BioRender.com. Source data

**Extended Data Fig. 7:**
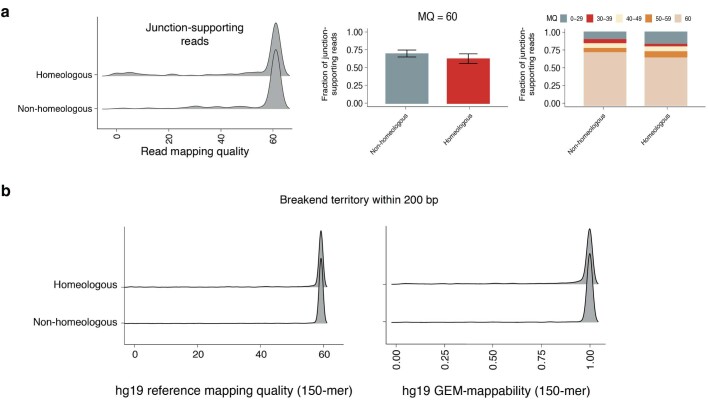
Support for homeologous junctions. **a**, Comparison of mapping quality for reads supporting homeologous and non-homeologous junctions. Left, density plot showing mapping quality for junction-supporting reads realigned to hg19, from a subsample of 500 non-homeologous junctions and 500 homeologous junctions. Middle, bar plot of the fraction of reads with high mapping quality (MQ = 60). Error bars represent 95% confidence interval on the Bernoulli trial parameter calculated using the fraction of non-homeologous (73,871/95,934) or homeologous (16,954/31,078) junction-supporting reads that had MQ of 60. Right, bar plot of the fraction of reads stacked by intervals of mapping quality. The number of reads in each MAPQ range for homeologous junctions (31078 total) is: MAPQ 0–29: 12,025, MAPQ 30–39: 626, MAPQ 40–49: 676, MAPQ 50–59: 797, MAPQ 60: 16,954. The number of reads in each MAPQ range for homeologous junctions (95,934 total) is: MAPQ 0–29: 19,201, MAPQ 30–39: 1,052, MAPQ 40–49: 868, MAPQ 50–59: 942, MAPQ 60: 73,871. **b**, Left, reference 150-mer BWA mapping quality in the neighbourhood of homeologous and non-homeologous break ends. Reference mapping quality determined by realigning sliding window of 150-mers from hg19 stepping by 1 base to hg19 and averaging across each base pair. Right, plot of alternate mappability scores calculated as the average of the reciprocal of the number of unique locations that each 150-mer overlapping a break-end-associated base pair aligns. Source data

To better assess the burden of SSA in LR WGS profiles, we developed and validated an algorithm (Methods and Supplementary Note 8) to detect runs of homeology, or 80% or higher sequence identity, near somatic break ends (Fig. 4a). This algorithm identified a peak of homeology around 50 bp (Fig. 4b), yielding 138 junctions with homeology greater than 10 bp across 46 LR WGS samples. Notably, most of these homeologous junctions were also detected with high efficiency in short-read WGS (Extended Data Fig. 7a,b and Supplementary Note 9), indicating that our analysis of homeology could be applied to the full short-read WGS BOPP dataset.

Analysis of 583 tumour–normal pairs (BRCA1d, BRCA2d or HRP) revealed that 1,248 of 49,561 (2.5%) junctions were homeologous, with a distribution that mirrored LR WGS (Fig. 4c). Although the median homeology length among these junctions was 40 bp, we observed tracts as long as 128 bp. We next asked which classes of simple or complex variants contained homeologous junctions. Comparing distributions across genotypes revealed that BRCA2d tumours had a significantly higher burden and fraction of larger (more than 1 kbp) homeologous deletions relative to BRCA1d (RR = 3.93, *P* = 4.2 × 10^−4^, Wald test on gamma-Poisson regression) and HRP cancers (RR = 3.28, *P* = 8.37 × 10^−6^, Wald test on gamma-Poisson regression; Fig. 4d). Although we also observed homeologous break ends in other SV classes, the burden of these events did not correlate with HR-proficiency status (data not shown). Notably, the median size of homeologous deletions (around 10 kbp; Fig. 4e) was consistent with the length of end resection that is known to occur in BRCA2d cells^31,32^, supporting the role of SSA as a backup repair pathway in human BRCA2d tumours (Fig. 4f).

## SV features improve HRD subclassification

**Extended Data Fig. 8:**
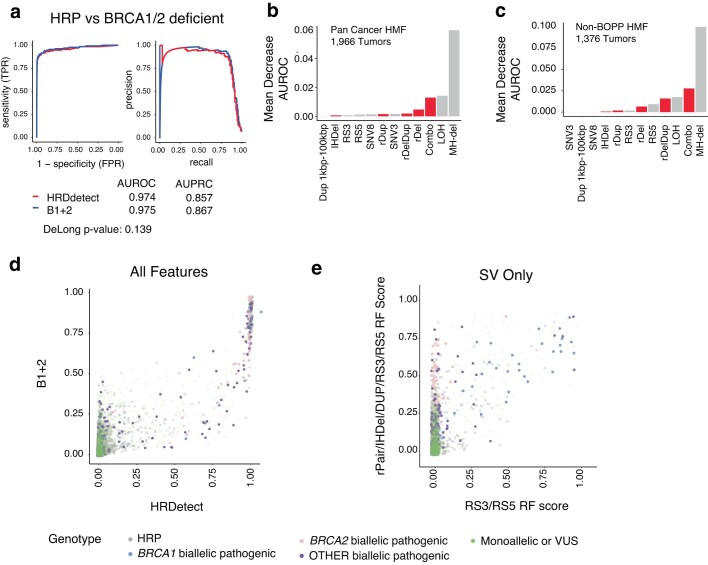
Comparing the performance of B1+2 and HRDetect. **a**, ROC curves and PRCs comparing B1+2 and HRDetect performance on detecting HR deficiency (BRCA1d or BRCA2d) in an independent (HMF) test set. *P* value denotes comparison of AUROC by DeLong test. **b**,**c**, B1+2 feature importance for detecting HR deficiency in (**b**) an independent pan-cancer WGS dataset and (**c**) its non-BOPP subset . See Extended Data Fig. 1 and Methods for training and testing dataset summary. **d**, Scatter plot of B1+2 versus HRDetect scores across all 7,918 tumours, including those excluded from training and testing **e**, Scatter plot of scores from a random forest model trained only on SV features from the B1+2 (*y* axis) or HRdetect classifier (*n* = 7,918). Source data

Having identified SV footprints of backup repair that are specific to BRCA1d and BRCA2d cancers, we next sought to understand whether these features could improve pan-cancer HR-deficiency classification. To assess the predictive value of the SV features highlighted in our study, we built a pan-cancer HR-deficiency classifier B1+2, which augments the six features used by HRDetect with the five highlighted in our study (1–100-kb tandem duplications, rDups, rDelDups, rDels and homeologous deletions; Methods and Supplementary Note 10). We trained the classifier on 62 BRCA1d, 64 BRCA2d and 2,536 HRP pan-cancer cases built from our BOPP and MSKCC datasets and additional publicly available samples (Extended Data Fig. 1 and Supplementary Note 11). When applied to an independent test set of 1,966 BRCA1d, BRCA2d and HRP pan-cancer WGS profiles from the Hartwig Medical Foundation (‘HMF’ dataset), B1+2 showed a marginal improvement over HRDetect (B1+2 area under the receiver operator characteristic (AUROC) = 0.98, AUPRC = 0.87; HRDetect AUROC = 0.97, AUPRC = 0.86; Extended Data Fig. 8a). Indeed, as with HRDetect, classification was mainly driven by the fraction of indels with microhomology, yielding similar scores between the two algorithms (Extended Data Fig. 8b–d).

**Fig. 5:**
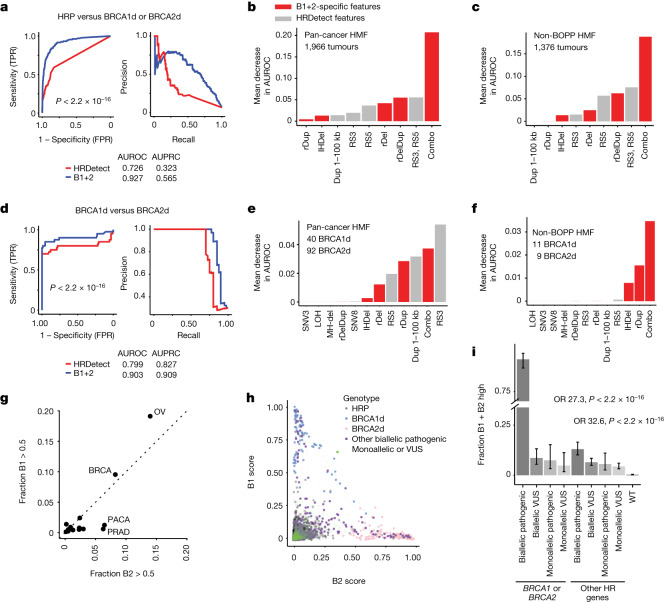
SV features distinguish between BRCA1 and BRCA2 deficiency. **a**, Receiver operating characteristic (ROC) curve and precision recall curve (PRC) comparing SV features highlighted in this study with those used in HRDetect for accurately classifying HR deficiency (either BRCA1d or BRCA2d). *P* values denote comparison of AUROC by two-tailed DeLong test. TPR, true positive rate. FPR, false positive rate. **b**,**c**, Importance of highlighted SV features in an independent pan-cancer WGS dataset (**b**)  and its non-BOPP subset (**c**). ‘Combo’ refers to a combination of the B1+2 classifier-specific SV features highlighted in this study. IHDel, SV deletion with inexact homology. **d**, ROC curves assessing B1+2 and a random forest classifier using only the six HRDetect features in predicting BRCA1d versus BRCA2d status. **e**,**f**, Feature importance for BRCA1d versus BRCA2d classification in an independent pan-cancer WGS dataset (**e**) and its non-BOPP subset (**f**). See Extended Data Fig. 1 and Methods for training and testing dataset summary. MH-del, short deletion with microhomology. **g**, Frequency of HR deficiency, as defined by either B1 or B2 score > 0.5, among common cancer types, including samples excluded from training and testing for harbouring VUSs or monoallelic variants (*n* = 7,918). BRCA, breast adenocarcinoma; OV, ovarian cancer; PACA, pancreatic adenocarcinoma; PRAD, prostate adenocarcinoma. **h**, B1 versus B2 scores in the B1+2 classifier. **i**, Fraction of cases that are B1 or B2 positive (score > 0.5) for cases with a rare biallelic germline or somatic mutation in additional (that is, not *BRCA1* or *BRCA2*) HR-pathway genes (*n* = 7,918 tumours). Error bars show 95% confidence interval on the Bernoulli trial parameter. *P* values and odds ratios obtained with Fisher’s exact test, without adjustment for multiple comparisons. WT, wild type. Source data

Given that HR deficiency is a disorder of DSB repair and altered genome structure, we next asked how well HR deficiency could be predicted solely on the basis of break-point-level structural genomic features. To assess this, we compared the performance of a random forest classifier trained using only the SV features in HRDetect (RS3 and RS5 signatures) and one trained with additional SV features specific to B1+2 (homeologous deletions, reciprocal pairs and 1–100-kbp simple duplications). We found substantially better performance (*P* < 2.2 × 10^−16^, DeLong test) with the B1+2-based SV classifier (AUROC = 0.93, AUPRC = 0.57, pan-cancer HMF) relative to the SV classifier based on HRDetect (AUROC = 0.73, AUPRC = 0.57; Fig. 5a). Although certain B1+2 classifier-specific SV features were individually relevant, the highest performance was observed when these features were used in combination (Fig. 5b,c). The performance improvement was most clearly attributable to the B1+2-specific SV features that recognized BRCA2d tumours (Extended Data Fig. 8e).

**Extended Data Fig. 9:**
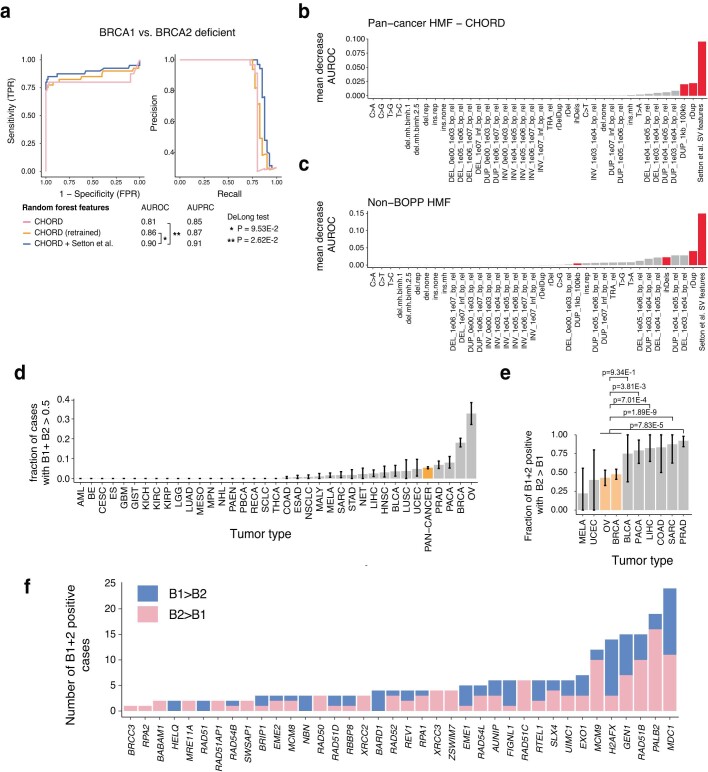
Distinguishing between BRCA1 and BRCA2 deficiency. **a**, ROC curves and PRCs for discriminating BRCA1d from BRCA2d tumours by CHORD and by a random forest trained on CHORD features augmented with the SV classes introduced in this manuscript. *P* values obtained by two-tailed DeLong test. **b**,**c**, Feature importance for BRCA1 versus BRCA2 deficiency classification in (b) an independent pan-cancer WGS dataset and (c) its non-BOPP subset. See Extended Data Fig. 1 and Methods for training and testing dataset summary. **d**, Tumour type and pan-cancer prevalence of B1+2 positivity (*n* = 7,918 tumour samples). The fraction of B1+2 positive (B1 + B2 score > 0.5) cases per tumour type is shown on the left; only tumour types with at least 20 examples are shown. Orange bar denotes the pan-cancer B1+2 positivity rate. Error bars show 95% confidence interval on the Bernoulli trial parameter. **e**. The fraction of cases with B2>B1 out of the cases that were B1+2 positive (*n* = 7,918 tumour samples); only tumour types with at least five B1+2 positive examples are shown. Tumour types significantly enriched for B2 positivity relative to the reference class (breast and ovarian cancer, highlighted in orange) are indicated with stars. Error bars show 95% confidence intervals on the Bernoulli trial parameter and stars indicate relative enrichment of B2 positivity within each tumour type. *P* values were obtained by two-tailed Fisher’s exact test. **f**, Number of B1+2 positive (B1+ B2 score > 0.5) cases with B1>B2 or B2>B1 and harbouring biallelic or monoallelic and pathogenic or VUS variants in HR-associated genes (see main text and Methods). LOH = burden of large genomic segments harbouring loss of heterozygosity. HomeoDel = count of large deletions (>1 kbp) with homeology. del-MH = proportion of small deletions (<50 bp) with microhomology. RS3, RS5 = proportion of junctions with rearrangement signature 3 or 5 (ref. ^2^). SBS3, SBS8: COSMIC single base signature 3 and 8 (ref. ^60^). Source data

We next asked whether B1+2 could distinguish between BRCA1 and BRCA2 deficiency, which are distinct biological states, each with possibly distinct therapeutic vulnerabilities^34^. As HRDetect was not developed to address this task, we compared B1+2 to a random forest classifier trained on six HRDetect features (see above). We found that B1+2 substantially outperformed (AUROC = 0.90, AUPRC = 0.91) this HRDetect-like classifier (AUROC = 0.80, AUPRC = 0.83) in distinguishing BRCA1d from BRCA2d tumours (*P* = 0.005, DeLong test; Fig. 5d). B1+2 classifier-specific SV features were particularly important for making this distinction in non-BOPP cancers (Fig. 5e,f). We also performed similar comparisons to CHORD^4^ (Extended Data Fig. 9a–c and Supplementary Note 12). As B1+2 outputs the separate probability of BRCA1d (B1 score) and BRCA2d (B2 score), we could analyse the probability of BRCA1d or BRCA2d in the tumours called HR-deficient by the classifier (B1+2 positive, B1 + B2 score > 0.5). This analysis confirmed that prostate and pancreatic cancer HR deficiency is significantly enriched in the BRCA2d phenotype relative to breast and ovarian cancer, in which BRCA1 and BRCA2 deficiency are equally likely^35,36^ (Fig. 5g). Extending this analysis to non-BOPP samples, we found a lower rate (less than 5%) of HR deficiency, but with an increased bias toward BRCA2 deficiency (hepatocellular carcinoma and sarcoma; Extended Data Fig. 9d,e).

A major use of HR-deficiency genomic signatures is to uncover alternate mechanisms by which the HR pathway is inactivated and assess the pathogenicity of variants of uncertain significance (VUSs). Investigating B1+2 score distributions in cases that were excluded from our training and test data (Fig. 5h; including cases with monoallelic alterations and VUSs in *BRCA1* and *BRCA2* and/or other HR-pathway alterations; Supplementary Table 1), we found a significantly higher rate of B1+2 positivity across various strata of monoallelic and/or VUS cases (Fig. 5i and Supplementary Note 13), although this rate was substantially lower than that for cases with biallelic pathogenic alterations in *BRCA1* or *BRCA2* (95%). This included genes with distinct biases for BRCA1d (*BARD1* and *EME1*) versus BRCA2d (*PALB2* and *RAD51C*), consistent with their known roles in the HR pathway (Extended Data Fig. 9f). These results indicate that the B1+2 classifier could help to uncover and subclassify pathogenic alleles that are responsible for HR deficiency.

**Extended Data Fig. 10:**
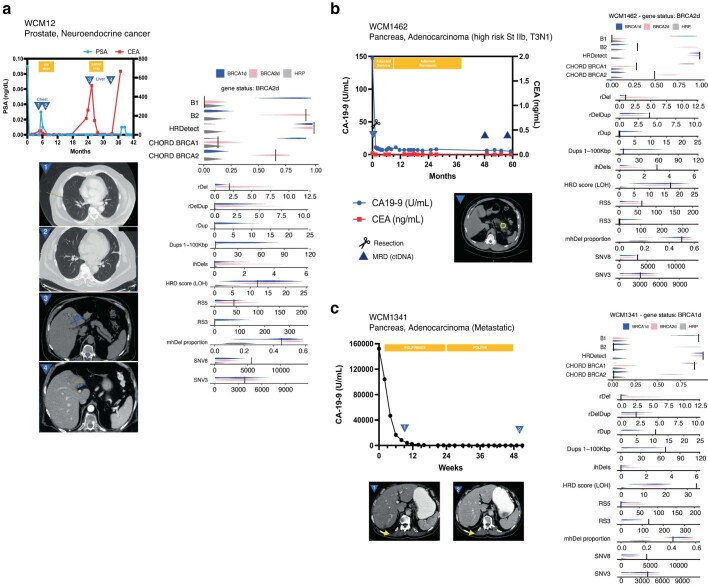
Clinical vignettes of patients with HRD tumours. **a**, Patient with BRCA2d neuroendocrine prostate cancer. Top left, prostate-specific antigen (PSA) and carcinoembryonic antigen (CEA) response kinetics. Time points 1–4 correspond to axial computed tomography images depicted on bottom right, illustrating favourable response to platinum-based chemotherapy. Right, classifier scores (top) and genomic features (bottom) for the highlighted patient (vertical lines) vs dataset-wide distributions stratified by genotype (violin plots; BRCA1d n = 102, BRCA2d n = 158, HRP n = 4360, HRP). **b**, Metastatic pancreas adenocarcinoma case with high B1 score (0.962), with CA19-9 response kinetics (left) and serial axial computed tomography (CT) (blue triangles and corresponding right panels) demonstrating excellent response to chemotherapy. **c**, High risk stage IIb pancreas adenocarcinoma case (WCM1462) with high B2 score (0.31), with CA19-9 and CEA response kinetics (top) and axial CT (bottom). LOH = burden of large genomic segments harbouring loss of heterozygosity. ihDels = count of large deletions (>1 kbp) with homeology. mhDel proportion = proportion of small deletions (<50 bp) with microhomology. RS3, RS5 = proportion of junctions with rearrangement signature 3 or 5 (ref. ^2^). SBS3, SBS8: COSMIC single base signature 3 and 8 (ref. ^60^).

To further assess the relevance of classifier results, we investigated clinical outcomes for three cases with high B1+2 scores among 80 WGS cases profiled at Weill Cornell Medicine (Methods). All three cases with adequate follow-up data showed favourable responses to platinum chemotherapies and/or PARP inhibition (Supplementary Fig. 1 and Extended Data Fig. 10). This included a B2-high (B2 = 0.912) de novo case of metastatic neuroendocrine prostate cancer with an atypical (20.7 months) extracranial complete response to first-line platinum doublet (cisplatin–docetaxel) therapy and a second complete response to platinum rechallenge. The other two cases showed survival that exceeded the expectation (less than one year) for tumours of this histology and stage. Although all three cases also showed high HRDetect and CHORD scores, B1+2 provided extra certainty in distinguishing between BRCA1d and BRCA2d (Extended Data Fig. 10).

## Discussion

Our LR WGS study provides one of the largest datasets so far of long-molecule whole-genome profiles in DNA-repair-deficient cancers. Long-molecule phasing allowed us to specifically link *trans* reciprocal pairs to large-scale chromosomal alterations. These results address a paradox in the field by providing a link between HR-deficiency-specific rearrangement patterns and megabase-scale cytogenetic phenotypes that are associated with HR deficiency^11,37,38^.

Long-molecule data also reveal specific scars of backup repair pathways in HRD cells, including SSA, which has long been thought to help to maintain genome stability in BRCA2d cells^31^. Although SSA has been extensively studied using induced DSBs in synthetic plasmid reporter systems, it has not previously been shown to be relevant to human BRCA2d cancer genomes. Our data also provisionally extend the relevance of a second repair mechanism, homology-independent replication restart (Fig. 3g and Extended Data Fig. 6g,h), which has been previously implicated in BRCA1-deficiency-associated tandem duplications^20^. Extension of this mechanism to reciprocal pairs is most strongly supported by the existence of translocations and large inversions (*trans* rDups and rDelDups) with substantial (1–100 kbp) DNA duplication at one or both junctions. The substantial (more than 50 bp) duplications seen at *trans* rDups and rDelDups cannot be explained by simple end-joining but imply a replication-coupled repair process.

In HRP cells, BIR restarts replication when stalled and/or collapsed forks create single-ended DSBs^20,22,39^. A RAD51–RAD52-independent variant of BIR called microhomology-mediated BIR (MMBIR) can repair single-ended DSBs by invading nearby DNA duplexes in the absence of homology^26,28^ and drive replication restart in HRD cells^23^. MMBIR intermediates are also exceptionally prone to template exchanges and crossovers, and thus provide the most plausible candidate for the genesis of reciprocal pairs as well as more complex SVs. In particular, factors that stabilize displacement loops and facilitate longer tracts of repair synthesis increase the likelihood of crossover products after BIR^20,22,40–42^, consistent with our observation that *trans* reciprocal SVs contain larger duplications than do their *cis* counterparts (Fig. 3a and Extended Data Fig. 6f).

The ultimate criterion by which to judge HR-deficiency classifiers is their ability to predict response to genotoxic therapy. Assessment of this hypothesis beyond a few vignettes (Extended Data Fig. 10) will require large retrospective analyses of clinically annotated and WGS-profiled cases or prospective clinical trials with WGS-based classifiers as an end-point. Furthermore, the improved ability to distinguish between phenotypes of BRCA1 and BRCA2 deficiency, previously also addressed by CHORD^4^, could inform future clinical trials that target BRCA1d- or BRCA2d-specific vulnerabilities^34^. As clinical WGS becomes cheaper and more practical, the routine implementation of approaches such as B1+2, which use more detailed features of BRCA1- and BRCA2-deficiency-specific SV patterns, might become an essential part of therapeutic decision-making.

## Methods

### Pan-cancer WGS data sources

GrCh37/hg19 BAM alignments for 2,489 primary tumour and matched normal whole-genome sequencing data were obtained as previously described^18^. In brief, 989 tumour–normal (T/N) pairs were obtained from The Cancer Genome Atlas (TCGA) Research Network (Genomic Data Commons at https://portal.gdc.cancer.gov/, accession: phs000178.v11.p8). Additional WGS data were obtained for 874 T/N pairs from the International Cancer Genome Consortium (ICGC) from multiple studies publicly available through the European Genome-phenome Archive (EGA; https://ega-archive.org). These cohorts include: 124 breast cancers^21^ (EGA: EGAS00001001178), 179 melanomas^43^ (EGA: EGAS00001001552), 49 lung adenocarcinomas^44^ (EGA: EGAS00001002801), 422 oesophageal adenocarcinomas^45^ (EGA: EGAD00001004417) and 100 malignant lymphomas (EGA: EGAD00001002123).

Additional BAMs for 121 T/N pairs from a pan-cancer cohort obtained as part of a New York City-based multi-institution collaborative research effort comprising the Memorial Sloan Kettering Cancer Center (MSKCC), New York University, Stony Brook University Hospital, Lenox Hill, Northwell Health, Columbia University, Montefiore, and Cornell, and led by the New York Genome Center, were included here and were previously described^18^. Study approval was obtained through a central institutional review board (IRB), Biomedical Research Alliance of New York, and by local IRBs, including Stony Brook University and Northwell Health. In addition, 55 prostate cancers that were previously published were obtained through dbGaP with accession phs000447.v1.p1 (ref. ^15^). BAMs for 80 T/N pairs were obtained from a collaborative precision oncology effort between the Weill Cornell Englander Institute for Precision Medicine (EIPM) and the New York Genome Center. This study was approved by an institutional review board (WCM IRB no. 1305013903). A total of 340 T/N pairs across 80 cases across longitudinally or spatially distinct biopsies from Barrett’s oesophagus tumours were obtained as part of a previous study^46^.

Call sets were obtained from 1,484 additional T/N pairs contributing additional primary tumour whole genomes from the Pan-Cancer Analysis of Whole Genomes Consortium^47^ (Extended Data Fig. 1, ‘PCAWG’ dataset, https://dcc.icgc.org/pcawg) and 3,957 T/N pairs from metastatic whole genomes from the Hartwig Medical Foundation (HMF, https://www.hartwigmedicalfoundation.nl/), which included germline, somatic SNV or indel, and somatic SV calls^48^ (Extended Data Fig. 1: ‘HMF’ dataset).

### MSKCC cohort

LR WGS and short-read WGS were performed on a cohort of 46 cases biopsied for ductal carcinomas of breast and found to have *BRCA1* (*n* = 28) or *BRCA2* (*n* = 18) mutations on clinical panel sequencing. These cases were collected under informed consent as part of a prospective biospecimen research protocol at the Memorial Sloan Kettering Cancer Center (MSKCC, MSKCC IRB no. 16–675). T/N pairs were profiled with Illumina short-read WGS and LR WGS (see below for protocol details). Raw sequencing data from these experiments have been made  available (see ‘Data availability’ section; Extended Data Fig. 1: ‘MSKCC’ dataset).

### Pipelines

Harmonized variant calling was performed on 2,489 T/N BAM file pairs by adapting previously described pipelines^18^. Additional details are provided below.

### SV calling

In brief, genome-wide, 200-bp binned tumour and normal read depth was calculated from alignments and corrected for GC and mappability biases (https://github.com/mskilab-org/fragCounter). Somatic SV calls were obtained with SvAbA^49^ and filtered using a panel-of-normals (PON) comprising all germline SVs detected across 2,489 T/N pairs. Any somatic SV found within 500 bp of a junction within the germline SV PON with matching orientations was discarded. PCAWG consensus SVs and 200-bp binned tumour and normal read depths were obtained from PCAWG SV release 1.6 and the PCAWG data coordination centre.

HMF SV data were obtained from the Hartwig Medical Foundation through a data sharing agreement^48^. In brief, junction calls from GRIDSS^50^ and 1-kbp tumour/normal coverage ratios^17^ corrected for GC content were obtained for 3,957 T/N pairs.

### Genome graph analysis

High-confidence junctions, binned tumour-normal read depth ratios, and purity and ploidy estimates (see below) were used to perform junction balance analysis (JaBbA; github.com/mskilab-org/JaBbA) and generate balanced genome graphs for 7,918 and 46 cases in the pan-cancer WGS and MSKCC datasets, respectively. For a detailed treatment of the formulation behind JaBbA, see a previous report^18^. Heterozygous germline single-nucleotide polymorphism (SNP) data were used to infer allelic copy number after total copy number inference was performed genome-wide as described^18^.

### Purity and ploidy estimation

Across Hadi dataset cases, tumour purity and ploidy were estimated using ASCAT^51^. For the 46 cases from the MSKCC cohort, a manual review of purity and ploidy was conducted to enhance downstream genotyping accuracy; ultimately, alternative manual estimates of purity and ploidy from MSKCC were chosen for 4 out of 46 cases. For PCAWG and HMF datasets, purity and ploidy estimates were obtained from the respective PCAWG (https://dcc.icgc.org/releases/PCAWG/) and HMF (https://www.hartwigmedicalfoundation.nl/en/database/) portals^48^.

### LR WGS SV calling

For the LR WGS profiles generated from the MSKCC cohort of 46 cases, junctions called using LinkedSV^52^ were merged with SvAbA junctions called on the corresponding short-read WGS for each case. These were then input into JaBbA using short-read coverage profiles to generate genome graphs. Merging was performed using the gGnome R package (https://github.com/mskilab-org/gGnome) to determine junctions that were uniquely detected by LR WGS (LinkedSV).

### Analysis of gap segment topology

Gap segments were defined as short genomic segments joining reference-consecutive break ends, each belonging to distinct junctions and occurring on opposite strands. Each gap segment was additionally associated with a polarity (+ or −) based on the topology of junctions around the gap segment; (+) polarity corresponded to a gap segment with junctions directly attached to it, (−) polarity corresponded to a gap segment with junctions attached to the two segments to the left and right of the gap segment on the reference. The length threshold to define gap segments was visually chosen as 1 Mbp after inspection of a density plot of segments lengths across gap segment candidates satisfying the above topological criteria. This threshold was confirmed through the application of a background model, in which the gap segment candidate length distribution in each sample was fit with an exponential distribution and each gap segment candidate was assigned a *P* value according to the left tail of the exponential cumulative distribution function. Short (less than 1 Mbp) gap segment candidates were found to significantly deviate from expectation (false discovery rate (FDR) < 0.10) under this model.

Applying this definition, gap segments with shared junctions were next clustered together (applying ‘eclusters’ gGraph method in the gGnome R package) to identify and classify reciprocal clusters. Reciprocal clusters in which every junction was connected to two gap segments was labelled as ‘cyclic’. Reciprocal clusters were annotated with the number of cluster-associated junctions and gap segments. A reciprocal pair (rPair) is a special case of a cyclic reciprocal cluster that contains two gap segments of either orientation. rDups, rDels and rDelDups each contain two (+) gap segments, two (−) gap segments and one (+) and one (−) gap segment, respectively.

### Annotating known SV events

Classes of previously described^18^ simple and complex SV were annotated in balanced genome graphs derived by JaBbA for both the pan-cancer WGS (*n* = 7,918) and MSKCC datasets (*n* = 46). The following simple events were annotated within each graph: deletions, duplications, translocations, inversions and inverted duplications. The following complex events were annotated: breakage–fusion–bridge cycles, double minutes, tyfonas, chromoplexy, chromothripsis and TICs. Implementation of each event classifier can be found in the ‘events’ function in the gGnome R package.

### Variant calling and genotyping

For the 2,489 ‘Hadi’ dataset WGS T/N pairs (Extended Data Fig. 1), somatic mutation calls were generated with Strelka1 for SNVs and indels. Germline mutation calls were obtained with Strelka2 run on alignments from blood or adjacent normal samples and filtered to remove common variants above a population allele frequency of 1% (ExAC population: ftp.broadinstitute.org/pub/ExAC_release/release0.3.1/subsets/). SNVs and indels were filtered through a universal genome-wide mask for hg19 (https://github.com/lh3/CHM-eval) to remove artefacts due to low mappability, as described before^53^. All germline and somatic SNVs and indels were annotated with ClinVar status (ftp.ncbi.nlm.nih.gov:/pub/clinvar/vcf_GRCh37; database date 2022-07-30). The impacts of protein-coding SNVs and indels were also annotated through SnpEff (GRCh37.75 database). SNVs and indels were considered pathogenic if annotated as ‘pathogenic’ or ‘likely_pathogenic’ through ClinVar CLNSG or if their SnpEff annotation fell within the following classes: ‘frameshift variant’, ‘start_lost’, ‘stop_gained’, ‘stop_lost’, ‘splice_acceptor_variant’ or ‘splice_donor_variant’. ClinVar annotation took precedence over SnpEff.

LOH was determined by allele-specific copy number (CN) using allele counts across germline heterozygous SNP sites. Specifically, LOH was called in regions in which minor allele CN = 0 and major allele CN > 0, using allelic copy number as inferred from short-read sequencing data (see ‘Junction balance analysis’). Similarly, homozygou deletions (homdels) were called in regions in which total copy number (sum of major and minor allele CN) = 0.

Genotype was determined across samples for 48 HR-related genes (Supplementary Table 1), including *BRCA1*, *BRCA2*, *PALB2* and *RAD51C*. Eleven of these genes were highlighted in a previous study^54^. Biallelic loss was called for genes if they contained any of the following: (1) two or more germline and/or somatic pathogenic mutations (including SNVs, indels and SVs); (2) one germline or somatic pathogenic mutation along with LOH; or (3) a homozygous deletion. Within the MSKCC cohort, 22 cases were found to have biallelic loss of *BRCA1*, 14 cases were found with biallelic loss of *BRCA2*, and one case was found with biallelic loss of both *BRCA1* and *BRCA2*.

For PCAWG dataset cases, somatic SNV and indel calls were obtained from ICGC (2016 data freeze), and annotated driver mutations were obtained from the PCAWG consortium^47^. HMF provided the following for cases in their dataset: germline SNVs and indels (through GATK HaplotypeCaller), somatic SNVs and indels (through Strelka1 and annotated by SnpEff).

### Short-read WGS

Short-read WGS for the 46 MSKCC donors was performed at the New York Genome Center to a target of 80× tumour and 40× normal coverage. Library preparation from genomic DNA for these new cases was performed using the NEBNext Ultra II End Repair/dA-Tailing Module, NEBNext Ultra Ligation Module (New England Biolabs) and KAPA Dual-Indexed Adapter Kit (Roche) following the manufacturers’ protocols. Quality control was performed on the finished libraries with the Agilent 2100 Bioanalyzer on the High Sensitive DNA Chip platform (Agilent Technologies) and KAPA Library Quantification Kit (Roche). Quality control determined that libraries contained an average peak height (fragment size) of at least 400 bp. Libraries were sequenced on an Illumina NovaSeq 6000 System (Illumina) to generate paired-end 2 × 150-bp reads. Reads were aligned to the GRCh37/hg19 reference using Burrows–Wheeler aligner software^55^, bwa mem, 0.7.10-r789). Read post-processing was done in accordance with best practices for post-alignment data processing with Picard tools (https://broadinstitute.github.io/picard/) to mark duplicates, the GATK (v.2.7.4) (https://gatk.broadinstitute.org/hc/en-us) IndelRealigner module and GATK base quality recalibration.

### LR WGS

Each of the 46 *BRCA1-* or *BRCA2*-mutant cases in the MSKCC cohort was subjected to additional LR WGS. High-molecular-weight (HMW) genomic DNA (gDNA) was extracted using a Qiagen MagAttract HMW DNA Kit (Qiagen) according to the suggested protocol. In brief, approximately 1–2 million cells were obtained from each frozen tissue sample and lysed, and HMW gDNA was captured by magnetic particles. Then the magnetic particles with HMW gDNA were washed in wash buffer and eluted in EB Buffer (10 mM Tris-HCl, pH 8.5). The HMW gDNA had a mode length of 50 kbp and max length 200 kbp, as estimated on a separate 75-V pulse-field gel electrophoresis using a BluePippin 5–430-kbp protocol (Sage Science). LR WGS library preparation was performed using a Chromium Genome Library Kit v2 (10X Genomics) following the Chromium Genome Reagent Kits v2 User Guide. In brief, 1 ng of extracted HMW gDNA was used to prepare a library, with an average fragment length of 625 bp (ranging from 300 to 2,000 bp, measured with the Agilent Bioanalyzer High Sensitivity DNA Chip). Quality control for the finished libraries was performed as above for the general WGS library preparation. The prepared libraries were sequenced on an Illumina NovaSeq 6000 Sequencing System (Illumina) with an S4 flow cell, to an average read depth of about 33×. All linked reads were aligned to GRCh37/hg19 with the EMerAld aligner (v.0.6.2)^56^. Germline haplotypes were obtained from Strelka2 germline SNV calls processed using HapCut2 (https://github.com/vibansal/HapCUT2; ref. ^57^).

### Phasing rearranged haplotypes with LR WGS

Our specific goal in somatic phasing was to distinguish SVs that placed both reciprocal junctions on the same molecule (*cis*) from those that placed junctions at distant loci (*trans*, including distinct derivative chromosomes) in the cancer genome (Extended Data Fig. 3a). Somatic phasing is distinct from parental phasing, which determines whether reciprocal break ends arose on the same or distinct parental homologue.  Namely, break ends that arise on the same parental homologue (germline *cis* phase) can end up on distinct derivative chromosomes (somatic *trans* phase).

We approached somatic phasing by assessing LR WGS molecule support for derivative rearranged haplotypes. Derivative rearranged haplotypes can be deconvolved from junction-balanced genome graphs as walks^18^. Walks were derived from JaBbA graphs on the MSKCC cohort for which both LR and short-read WGS were available using the ‘walks’ gGraph method in the the gGnome package (https://github.com/mskilab-org/gGnome). Barcoded reads were matched against each possible walk within a 100-kbp window of the junctions to be phased (gGnome score.walks function). The walk (or set of walks) that carried the largest number of junction-supporting barcodes was considered the likeliest haplotype explaining the rearrangement.

Specifically with respect to the rDup and rDelDup patterns, two sets of possible derivative haplotypes exist: *cis* and *trans*. *Cis* haplotypes for rDup or rDelDup patterns are walks that contain both involved rearrangements consecutively, or, in other words, contain a single segment that is flanked by both junctions. *Trans* haplotypes are two separate walks that each contain one of the two rDup or rDelDup rearrangements and would have to exist simultaneously, and thus are considered as a single set of walks. Junction-supporting LR barcodes were counted for each possible walk across every rDup or rDelDup instance in the MSKCC cohort. To assess whether the rDup or rDelDup patterns existed with the junctions in *cis* or *trans*, walk-supporting LR barcode counts were compared among the individual *cis* walks and the *trans* walks summed together. The walk (or set of walks in the *trans* case) was taken to be the derivative haplotype underlying the rDup or rDelDup pattern. Haplotypes were also validated by visually assessing the barcode sharing patterns for each rDup or rDelDup present in the dataset to confirm the haplotype as labelled by this heuristic.

### Imputing short-read-sequencing reciprocal pair haplotypes

To impute the haplotype phase of reciprocal pairs identified by short-read WGS, we applied a threshold of 3.5 to the log_10_ gap length. Specifically, for rDup reciprocal pairs, the imputed haplotype phase was *cis* if the minimum of the two log_10_ gap lengths was less than 3.5, and *trans* otherwise. For rDelDup pairs, the imputed haplotype phase was *cis* if the log_10_ length of the (+)-polarity gap was less than 3.5, and *trans* otherwise. The imputed phase of all rDel pairs was *trans* because this is the only phase possible given the junction topology.

### Sequence homeology

‘Homeology’ refers to approximate (higher than 80%) similarity between a pair of genomic sequences. To assess sequences at junction-associated break ends, we applied a sliding bin approach. For every position within a 200-bp window around each break end pair, a 41-bp bin centred at the base was queried for the corresponding hg19 reference sequence. All pairs of 41-bp bins within each junction-associated 200-bp window were then aligned to one another to construct a 200-by-200 matrix of Levenshtein edit distances. The distance matrix was the converted to a similarity matrix (Fig. 4a heat map) in which each entry *ij* indicates the sequence similarity, calculated as (1 − Levenshtein edit distance)/41, between a pair of 41-mers corresponding to bins *i* and *j* in the junction-associated window. The matrix was then converted to a binary bitmap image in which each pixel denoted sequence similarity of >0.8.  Connected components of pixels in the image were annotated with the Pearson’s correlation of the associated pixel indices, which was used as a measure of linearity of the pattern. Each junction was then annotated with the size (in pixels) of the largest connected component with a linearity of at least *r*^2^ > 0.9. This value thus represents the longest contiguous stretch of bases with at least 80% sequence similarity. This procedure was run using the ‘homeology’ function in  the GxG R package (see ‘Code availability’).

Discordant and split reads supporting junctions with homeology were realigned to hg19 using bwa mem (implemented using an R wrapper in the package RSeqLib), to obtain uniform mapping quality scores for those cases containing junction homeology within the in-house dataset in which alignments were present. Reference mappability was determined using two orthogonal means. In the first, sliding 150-mers stepping by one base were queried across hg19 and aligned to the full reference using bwa mem to determine mapping quality scores. Average mapping quality was determined for each base for hg19. The second method used GEM mappability score with a sliding 150-mer across hg19 as described previously^58^.

### Mutational signatures

Mutational signatures were derived from the signature.tools.lib R package suite for implementing the HRDetect algorithm^2^. In brief, SNV signatures were deconvolved using the known signature weights from COSMIC SNV signature version 2 (https://cancer.sanger.ac.uk/signatures/signatures_v2/, available through the signature.tools.lib R package^59^) with an implementation of non-negative least squares (‘SignatureFit’ function from the signature.tools.lib package). With the same approach, JaBbA-derived SVs were classified into the 32 SV types on the basis of size, topology and junction clustering as previously described^21^, and were fit to rearrangement signatures derived from 560 breast cancers. Microhomology in small deletions was searched in 3′ flanking sequence for up to 25 bases. The HRD-LOH index was determined by the number of segments per genome larger than 15 Mbp (but under the span of an entire chromosome) containing LOH.

### Classifying HR, BRCA1 and BRCA2 deficiency

To build classifiers distinguishing overall HR deficiency, BRCA1 deficiency and BRCA2 deficiency, random forests (RFs; from the randomForest R package) were trained on a dataset of pan-cancer primary tumours consisting of 62 BRCA1d, 66 BRCA2d and 2,536 controls that were confidently HRP (lacking CLINVAR pathogenic, CLINVAR VUS, truncating or missense mutation in *BRCA1*, *BRCA2*, *RAD51*, *RAD51B*, *RAD51C*, *RAD51D* and *PALB2* and LOH in *BRCA1* or *BRCA2*). The following six features were counted for each case using the R package signature.tools.lib: COSMIC SNV signatures 3 and 8; rearrangement signatures 3 and 5; HRD-LOH index; and proportion of deletions with microhomology. rDups, rDels and rDelDups were also counted after annotation on each genome graph.

To evaluate the performance of RFs, ROC curves and corresponding AUROCs were computed on an independent test set of pan-cancer metastatic tumours (HMF dataset, Extended Data Fig. 1) consisting of 40 BRCA1d, 92 BRCA2d and 1,834 HRP controls using the pROC R package (v.1.18.0, https://cran.r-project.org/web/packages/pROC/). Feature importance was determined by resampling the test set across 30 bootstraps with permutation. The decrease in accuracy after permuting each feature on the test set was calculated.

In the following two comparisons, classifier skill to discriminate overall HR deficiency from HR proficiency was analysed by using the full (Hadi, MSKCC, and PCAWG) training set and evaluating the resulting models on the full (HMF) test set (Extended Data Fig. 1). An SV-only RF was trained on rDups, rDels, rDelDups, homeologous deletions, duplications with length 10–100 kbp, RS3 and RS5 as features and compared against an RF trained on rearrangement signatures 3 and 5 as features to compare the efficacy of the classes of SVs described in this manuscript against the established SV types previously used in HRDetect^2^. A full RF consisting of currently described features and previously established features (rDups, rDels, rDelDups, homeologous deletions, duplications with length 10–100 kbp, RS3, RS5, MH-dels, SNV3, SNV8 and LOH score) was trained and then tested against the published HRDetect model (consisting of MH-dels, SNV3, SNV8, RS3, RS5 and LOH score as features) using ROC curves and feature importance metrics. HRDetect scores were obtained by running the function ‘applyHRDetectDavies2017’ from the signature.tools.lib R package on a feature matrix composed of test samples.

The third comparison evaluated classifier skill to discriminate BRCA1 deficiency from BRCA2 deficiency. For this test, the full RF trained with current and previous features was used to compare against a RF trained with HRDetect-only features. In contrast to the above, ROC and feature importance evaluation were performed on only the 40 BRCA1d and 92 BRCA2d cases from the test dataset (Extended Data Fig. 1).

### Statistical information

All statistical analysis was performed as stated in the figure legends using the R programming language (v.4.0.2). *P* values obtained that are smaller than 2.2 × 10^−16^ are not accurately estimated in R and are listed as such (‘*P* < 2.2 × 10^−16^’). Generalized linear modelling was performed using the ‘glm’ or ‘glm.nb’ function from the stats or MASS R packages. Wilcoxon rank-sum testing was performed using the ‘wilcox.test’ function from the stats R package. Fisher’s exact test was performed using the function ‘fisher.test’ from the stats R package. ROC curves were generated using the function ‘roc’ from the R package ‘pROC’. Comparison of ROC curves was done using the function ‘roc.test’ from the R package ‘pROC’ with the argument ‘method = ‘delong’. Statistical methods were not used to predetermine sample size.  The study design did not involve blinding or randomization. 

### Reporting summary

Further information on research design is available in the Nature Portfolio Reporting Summary linked to this article.

## Online content

Any methods, additional references, Nature Portfolio reporting summaries, source data, extended data, supplementary information, acknowledgements, peer review information; details of author contributions and competing interests; and statements of data and code availability are available at 10.1038/s41586-023-06461-2.

## Supplementary information

Supplementary Information This file contains Supplementary Notes; Supplementary Table 1; Supplementary Figure 1 and Supplementary References.

Reporting Summary

## Data Availability

The datasets generated for the current study include the WGS and LR WGS data for 46 *BRCA1* and *BRCA2*-mutated cases (see ‘MSKCC cohort’) have been deposited at the European Genome-phenome Archive (EGA), which is hosted by the European Bioinformatics Institute (EBI) and the Centre for Genomic Regulation (CRG), under accession number EGAD00001010326. Further information about EGA can be found at https://ega-archive.org (the European Genome-phenome Archive of human data consented for biomedical research). Processed data and an associated notebook for generating the main and Extended Data figure panels are provided as a GitHub repository (https://github.com/mskilab/setton_hadi_choo_2023). Source data are provided with this paper.

## Source data

Source Data Fig. 1
Source Data Fig. 2
Source Data Fig. 3
Source Data Fig. 4
Source Data Fig. 5
Source Data Extended Data Fig. 2
Source Data Extended Data Fig. 3
Source Data Extended Data Fig. 4
Source Data Extended Data Fig. 5
Source Data Extended Data Fig. 6
Source Data Extended Data Fig. 7
Source Data Extended Data Fig. 8
Source Data Extended Data Fig. 9

## Code availability

Executable notebook code spanning all key analyses across main and Extended Data figure panels is provided as a GitHub repository (https://github.com/mskilab/setton_hadi_choo_2023). Analyses were performed using R v.4.0.2 with R packages available from CRAN (https://cran.r-project.org/). The following lists R packages developed by authors to perform the described analyses. Genome-wide coverages for samples for which a BAM alignment was present were calculated with the fragCounter R package (https://github.com/mskilab-org/fragCounter). Fitting of junction-balanced genome graphs was carried out using the JaBbA R package^18^ (https://github.com/mskilab-org/jabba). Analysis of junction links and link clusters as well as classification of complex event types within each genome graph was performed with the function ‘eclusters’ in the package gGnome (https://github.com/mskilab-org/gGnome). Walk deconvolution on genome graphs was also performed using gGnome. LR WGS barcodes supporting junctions were queried using the ‘score.walks’ function in the skitools R package (https://github.com/mskilab-org/skitools). Visualization of genomic tracks were made with the gTrack R package (https://github.com/mskilab-org/gTrack). Analysis of sequence homeology across junction break ends is implemented with the function ‘homeology’ in the package GxG (https://github.com/mskilab-org/GxG). Custom tools for miscellaneous data manipulation tasks were implemented using the package khtools (https://github.com/kevinmhadi/khtools).
