# Supplementary material for: Long-molecule scars of backup DNA repair in BRCA1- and BRCA2-deficient cancers
Source: Nature. 2023 Aug 16;621(7977):129–37. doi: 10.1038/s41586-023-06461-2 (PMC10482687; doi:10.1038/s41586-023-06461-2)
Supplement: Supplementary file 1 — This file contains Supplementary Notes; Supplementary Table 1; Supplementary Figure 1 and Supplementary References. [file 41586_2023_6461_MOESM1_ESM.pdf]

nature portfolio

https://doi.org/10.1038/s41586-023-06461-2

Supplementary information

Long-molecule scars of backup DNA repair in BRCA1- and BRCA2-deficient cancers

In the format provided by the authors and unedited

Supplementary Information

Table of Contents

Supplementary Notes 2
Supplementary Table 1 4
Supplementary Figure 1 6
Supplementary References 6

Supplementary Notes

1. In order to confidently identify BRCA1d, BRCA2d, and HR-proficient cases in the BOPP dataset we required biallelic inactivation of *BRCA1* or *BRCA2* for a tumor to be classified as BRCA1d (n=24) or BRCA2d (n=36) respectively[1]. We also identified 487 HR proficient BOPP samples that lacked pathogenic or rare variants in any HR-associated gene (e.g. *BRCA1, BRCA2, PALB2*, *RAD51C*; see **Supplementary Table 1** for full list). We excluded the remaining 432 BOPP cases, which comprised tumors with monoallelic alterations and/or variants of unknown significance (VUSs) in *BRCA1* or *BRCA2* or mutations in other HR-associated genes.

2. Analysis of left-out BOPP samples ('Validation' dataset, **Extended Data Fig. 1**, 15 BRCA1d, 13 BRCA2d, 236 HR-proficient, n = 264 tumors) revealed significantly higher burdens of rDups in BRCA1d (μ = 6.87 per case) relative to BRCA2d (μ = 1.08, P = 2.0E-7, RR = 9.90, Wald test on gamma-Poisson regression) and HR-proficient (μ = 0.13, P = 6.7E-14, RR = 44.81) tumors **(Fig. 1c, right)**. Conversely, rDels were significantly enriched in BRCA2d (μ = 1.92 per case, P = 7.3E-5, RR = 4.29) relative to HR-proficient (μ = 0.67) tumors **(Fig. 1d, right)**. Finally, both BRCA1d (P = 5.0E-9, RR = 8.18) and BRCA2d (P = 1.4E-9, RR = 6.55) tumors harbored significantly higher burdens of rDelDups (μ = 2.20 and 2.00 per case, respectively) than HR-proficient cancers (μ = 0.32, **Fig. 1e, right)**.

3. Re-analysis of 46 cases with WGS (see MSK cohort in **Extended Data Fig. 1**) identified 22 BRCA1d and 14 BRCA2d cases, as well as one double-deficient (BRCA1d and BRCA2d) case, based on the LOH and allelic status of pathogenic alterations in *BRCA1* and *BRCA2.* The remaining 9 cases were found to have only monoallelic mutations in *BRCA1* (n=5) *BRCA2* (n=4) upon WGS analysis and were excluded from BRCA1d, BRCA2d, and HRP comparisons.

4. To assess whether reciprocal pairs could be responsible for large scale rearrangements in HR-deficient cancers, we inferred their derivative chromosomal structure, or phase to distinguish between *cis* and *trans* rearrangement outcomes on the basis of LR WGS alignment patterns (**Fig. 2a, Extended Data Fig. 4a,** see Methods). Our benchmarking analyses showed that we were able to distinguish between *cis* vs. *trans* configurations with high accuracy (>98%) across the range of LR molecule sizes and locus structures observed in our data (**Extended Data Fig. 5a,** see Methods).

5. We observed that *cis* rDelDups commonly harbor short (10bp - 1Kbp) ectopic E segments, whereas *trans* rDelDups harbor longer (1kb - 100 Kbp) duplicated E segments (**Extended Data Fig. 6a**), mirroring the relationship between phase and duplication tract length observed among rDups.

6. The observation that *cis* vs *trans* reciprocal pairs display significant differences in duplication tract lengths suggested that simple thresholds on gap segment lengths could be used to impute reciprocal pair phase in short-read WGS. We found that a threshold of 3,162 bp on the length of the smaller (+) polarity segment gave approximately 73% specificity and 75% sensitivity for imputing rDup and rDelDup phase (**Extended Data Fig. 6c,** see Methods).

7. A non-specific genomic hallmark of HR-deficiency is LOH[2]. We surmised that *trans* reciprocal pairs might be a source of LOH in two scenarios: (1) If a balanced translocation was followed by loss of one of the derivatives leading to telomeric LOH (e.g. through mis-segregation in a subsequent mitosis) and (2) if a type II intrachromosomal reciprocal pair gave rise to a circular acentric segment resulting in an interstitial LOH (**Fig. 3e**). To assess whether reciprocal pairs may serve as LOH precursors, we assessed gap segment to telomere distances for interchromosomal reciprocal pairs and gap segment to gap segment distances for intrachromosomal pairs and found a close correspondence between these distributions and observed LOH length distributions (**Fig. 3f**). Although such megabase-scale alterations are not unique to HR-deficient cancers,

their quantitative enrichment in such genomes is the basis of several currently employed clinical assays of HR-deficiency[3]. Our data suggest that *trans* reciprocal pairs likely underpin a substantial fraction of such LOH events.

8. We exhaustively quantified the fraction of matched bases in an optimal pairwise alignment across pairs of sliding 20 bp bins in the vicinity of breakends. We used these results to build raster images of homeology, which we found to harbor horizontal stretches of pixels representing runs of >80% sequence similarity in the human genome reference (**Fig. 4a**). To identify these patterns automatically, we built a simple image analysis algorithm (see Methods) to quantify homeology run length for candidate junctions.

9. While only 33 of 133 of LR WGS-derived homeologous junctions were found in the matched short-read WGS tumor data, 99 of 136 (73%) homeologous junctions detected by short-read WGS showed robust LR WGS support upon reinspection (**Fig. 4a**, see Methods). In both short-read and LR WGS, homeologous junctions were supported by high mapping quality reads (>75% with MAPQ=60 (**Extended Data Fig. 7a)**. Additionally, comparison of reference sequence in the vicinity of breakends showed similar fractions of unmappable bases between homeologous and non-homeologous junctions (**Extended Data Fig. 7b)**. These results indicate that the sequence divergence between homeologous breakend sequences was sufficient for the joined sequences to be distinguishable using short-reads.

10. To assess the predictive value of the SV features highlighted in our study, we built a classifier augmenting the six features used by HRDetect (deletions with microhomology, mhDel; COSMIC single base signature 3 and 8, SBS3 and SBS8; rearrangement signatures 3 and 5, RS3 and RS5, loss of heterozygosity, LOH) with the five highlighted by our study (1-100 kb tandem duplications, rDups, rDelDups, and rDels, homeologous deletions). This yielded an 11 feature random forest classifier called B1+2 (see Methods).

11. We augmented our short-read-only WGS BOPP dataset with LR WGS breast cancer cases and 2,662 pan-cancer WGS profiles giving a pan-cancer training set of 62 BRCA1d, 64 BRCA2d, and 2,536 HR-proficient cases (**Extended Data Fig. 1**).

12. CHORD[4] is a recently published algorithm that also distinguishes between BRCA1 and BRCA2 deficiency. Training a random forest on CHORD features augmented with the five B1+2 SV features improved performance though to a lesser extent (**Extended Data Fig. 9a)**. However, these B1+2-SV features were still the most important for distinguishing BRCA1 vs BRCA2 deficiency in an augmented CHORD classifier (**Extended Data Fig. 9b-c)**.

13. Notably, we found a significantly higher prevalence (12-25%) of B1+2 positivity (score > 0.5) among cases harboring a monoallelic or VUS mutation in *BRCA1* or *BRCA2* (OR 27.3, P < 2.2E-16, Fisher's Exact Test) or any deleterious alteration in non-*BRCA*1 or *BRCA2* HR genes (OR 32.6, P < 2.2E-16) relative to HR-proficient (<1%) cases.

Supplementary Table 1: List of HR-related genes. Genes associated with the homologous recombination pathway were manually curated from the literature.

Curated list of HR-related genes
AUNIP
BABAM1
BARD1
BRCA1
BRCA2
BRCC3
BRIP1
EME1
EME2
EXO1
FAM175A
FIGNL1
GEN1
H2AFX
HELQ
MCM8
MCM9
MDC1
MRE11A
MUS81
NBN
PALB2
RAD50
RAD51
RAD51AP1
RAD51B
RAD51C
RAD51D
RAD52
RAD54B
RAD54L
RBBP8
REV1
RMI1
RMI2

RPA1
RPA2
RTEL1
SFR1
SHFM1
SLX1A
SLX1B
SLX4
SWSAP1
UIMC1
XRCC2
XRCC3
ZSWIM7

Supplementary Figure 1: Characteristics of HR-deficient cases Clinical, pathologic, and genomic characteristics of three HR-deficient cases WGS profiled at Weill Cornell Medicine.

	WCM12	WCM1341	WCM1462
OT	0.917	0.962	0.31
B1/B2	0.005/0.912	0.948/0.014	0.011/0.299
OT Features			
tib	0	2	4
qrdel	2	0	1
qrdup	0	11	0
ihdel	2	1	3
del.mh.prop	0.454636092	0.417004049	0.500568828
SNV3	3827.548275	4016.416882	3161.368673
SNV8	5585.546508	4614.282765	3284.159033
DUP_1kb_100kb	1	68	6
hrd	12	34	17
RS3	0	131.1445011	0
RS5	44.45349227	0	61.468631
HRD gene status	BRCA2 (fs)	gBRCA1 + LOH	gBRCA2
Tumor type	Prostate	Pancreas	Pancreas
Histology	Neuroendocrine	Adenocarcinoma	Adenocarcinoma
Primary/Metastatic (location)	Prostate (RALP)	Liver	Primary (resection)
Clinical status (at tissue sampling)	Hormone naïve	Metastatic	Stage pIIb, T3N1, lymphovascular and perineural invasion
Treatment	1° - cisplatin/docetaxel x5	1° - FOLFIRINOX -> FOLFIRI	Adjuvant: gemcitabine/cisplatin x 6 months
	2° - carboplatin/etoposide x4	2° - RT to liver, PARPi, Y90 ablation	Rucaparib maintenance x 19 months
	3° - carboplatin x4	3° - GemOx	Surveillance x 14+ months
		4° - ICM	
Response	CR1 - extracranial PFS1 20.7 months (resection of two solitary brain metastases)	1° - PR	No recurrence
	CR2 - PFS2 11.7 months	2° - PR	
	PR3 - PFS3 4.9 months	3° - PR	
OS	37.2 months	32 months	59+ months
Status	Deceased	Deceased	Alive, off therapy x 21 months, NED on scans and via ctDNA (MRD-)

RALP - robotic-assisted laparoscopic prostatectomy; FOLFIRINOX - 5-FU/Leucovorin/irinotecan/oxaliplatin; FOLFIRI - 5-FU/leucovorin/irinotecan; PARPi - PARP inhibitor; GemOx - gemcitabine/oxaliplatin; ICM - cisplatin/irinotecan/mitomycin C; CR - complete response; PR - partial response; NED - no evidence of disease; MRD- - minimal residual disease; PFS - progression-free survival; OS - overall survival

Supplementary References

1. Riaz, N. *et al.* Pan-cancer analysis of bi-allelic alterations in homologous recombination DNA repair genes. *Nat. Commun.* **8**, 857 (2017).
2. Popova, T. *et al.* Ploidy and large-scale genomic instability consistently identify basal-like breast carcinomas with BRCA1/2 inactivation. *Cancer Res.* **72**, 5454–5462 (2012).
3. Marquard, A. M. *et al.* Pan-cancer analysis of genomic scar signatures associated with homologous recombination deficiency suggests novel indications for existing cancer drugs. *Biomark Res* **3**, 9 (2015).
4. Nguyen, L., W M Martens, J., Van Hoeck, A. & Cuppen, E. Pan-cancer landscape of homologous recombination deficiency. *Nat. Commun.* **11**, 5584 (2020).
