# Supplementary material for: Long-molecule scars of backup DNA repair in BRCA1- and BRCA2-deficient cancers
Source: Nature. 2023 Aug 16;621(7977):129–37. doi: 10.1038/s41586-023-06461-2 (PMC10482687; doi:10.1038/s41586-023-06461-2)
Supplement: Supplementary file 2 — Reporting Summary [file 41586_2023_6461_MOESM2_ESM.pdf]

nature portfolio

Corresponding author(s): Marcin Imielinski

Last updated by author(s): May 16 2023

Reporting Summary

Nature Portfolio wishes to improve the reproducibility of the work that we publish. This form provides structure for consistency and transparency in reporting. For further information on Nature Portfolio policies, see our Editorial Policies and the Editorial Policy Checklist.

Statistics

For all statistical analyses, confirm that the following items are present in the figure legend, table legend, main text, or Methods section.

n/a	Confirmed	
☐	☒	The exact sample size (*n*) for each experimental group/condition, given as a discrete number and unit of measurement
☐	☒	A statement on whether measurements were taken from distinct samples or whether the same sample was measured repeatedly
☐	☒	The statistical test(s) used AND whether they are one- or two-sided<br>*Only common tests should be described solely by name; describe more complex techniques in the Methods section.*
☐	☒	A description of all covariates tested
☐	☒	A description of any assumptions or corrections, such as tests of normality and adjustment for multiple comparisons
☐	☒	A full description of the statistical parameters including central tendency (e.g. means) or other basic estimates (e.g. regression coefficient) AND variation (e.g. standard deviation) or associated estimates of uncertainty (e.g. confidence intervals)
☐	☒	For null hypothesis testing, the test statistic (e.g. *F*, *t*, *r*) with confidence intervals, effect sizes, degrees of freedom and *P* value noted<br>*Give P values as exact values whenever suitable.*
☒	☐	For Bayesian analysis, information on the choice of priors and Markov chain Monte Carlo settings
☐	☒	For hierarchical and complex designs, identification of the appropriate level for tests and full reporting of outcomes
☐	☒	Estimates of effect sizes (e.g. Cohen's *d*, Pearson's *r*), indicating how they were calculated

Our web collection on statistics for biologists contains articles on many of the points above.

Software and code

Policy information about availability of computer code

Data collection	No code used for data collection.
Data analysis	Short reads were aligned to the GRCh37/hg19 reference using Burrows-Wheeler aligner software58, bwa mem, 0.7.10-r789. Read post-processing was done in accordance with best practices for post-alignment data processing with Picard tools (https://broadinstitute.github.io/picard/) to mark duplicates, the GATK (v.2.7.4) (https://gatk.broadinstitute.org/hc/en-us) IndelRealigner module, and GATK base quality recalibration. All linked-reads were aligned to GRCh37/hg19 with the EMerAld aligner (v0.6.2). Germline haplotypes were obtained from Strelka2 germline SNV calls processed using HapCut2 (github.com/vibansal/HapCUT2). SNV signatures were deconvolved using the known signature weights from COSMIC SNV signature version 2 (https://cancer.sanger.ac.uk/signatures/signatures_v2/, available through signature.tools.lib R package with an implementation of non-negative least squares ("SignatureFit" function from the signature.tools.lib package). To evaluate performance of random forests, receiver-operating characteristic (ROC) curves and corresponding areas under the curve (AUCs) were computed using the pROC R package (v1.18.0, https://cran.r-project.org/web/packages/pROC/). Generalized linear modeling was performed using "glm" or "glm.nb" function from the stats or MASS R package. Wilcoxon rank sum testing performed using "wilcox.test" function from the stats R package. Fisher's exact test was performed using the function "fisher.test" from the stats R package. Receiver-operator curves (ROC) were generated using the function "roc" from the R package "pROC". Comparison ROC curves was done using the function "roc.test" from R package "pROC" with argument "method = 'delong'".<br><br>Analyses were performed using R-4.0.2 with R packages available from CRAN (https://cran.r-project.org/). The following lists R packages developed by authors to perform the described analyses. Genome-wide coverages for samples for which a BAM alignment was present were calculated with the fragCounter R package (github.com/mskilab/fragCounter). Fitting of junction-balanced genome graphs was carried out using JaBbA R package (github.com/mskilab/jabba) (Hadi et al. 2020). Analysis of junction links and link clusters as well as classification of complex event types within each genome graph was performed with the function "eclusters" in the package gGnome (github.com/mskilab/

gGnome). Walk deconvolution on genome graphs was also performed using gGnome. l0X LR barcodes supporting junctions were queried using the "score.walks" function in the skitools R package (github.com/mskilab/skitools). Visualization of genomic tracks were made with the gTrack R package (github.com/mskilab/gTrack). Analysis of sequence homeology across junction breakends is implemented with the function "homeology" in the package GxG (github.com/mskilab/GxG). Custom tools for miscellaneous data manipulation tasks were implemented using the package khtools (github.com/kevinmhadi/khtools).

For manuscripts utilizing custom algorithms or software that are central to the research but not yet described in published literature, software must be made available to editors and reviewers. We strongly encourage code deposition in a community repository (e.g. GitHub). See the Nature Portfolio guidelines for submitting code & software for further information.

Data

Policy information about availability of data

All manuscripts must include a data availability statement. This statement should provide the following information, where applicable:

- Accession codes, unique identifiers, or web links for publicly available datasets
- A description of any restrictions on data availability
- For clinical datasets or third party data, please ensure that the statement adheres to our policy

The datasets generated for the current study include the WGS and 10X linked-read sequencing data for the 46 BRCA1&2-mutated cases (see Linked-read whole genome sequencing cohort) have been deposited at the European Genome-phenome Archive (EGA), which is hosted by the European Bioinformatics Institute (EBI) and the Centre for Genomic Regulation (CRG), under accession number EGAD00001010326. Further information about EGA can be found at https://ega-archive.org (the European Genome-phenome Archive of human data consented for biomedical research).The datasets generated for the current study include the WGS and l0X linked-read sequencing data for the 46 BRCA1&2-mutated cases (see Linked-read whole genome sequencing cohort) are available for download under NCBI BioProject accession: PRJNA746293.

Research involving human participants, their data, or biological material

Policy information about studies with human participants or human data. See also policy information about sex, gender (identity/presentation), and sexual orientation and race, ethnicity and racism.

Reporting on sex and gender	Cancer genomes were included for analysis in this study irrespective of their sex or gender.
Reporting on race, ethnicity, or other socially relevant groupings	Cancer genomes were included for analysis in this study irrespective of race, ethnicity, or other socially relevant groupings.
Population characteristics	Primarily European ancestry cancer genomes, see Extended Data Figure 1 for additional cohort details.
Recruitment	Consecutive breast cancer genomes with germline BRCAl/2 alterations (consented to MSK IRB 06-107, 12-245) were included in LR-sequencing cohort. Additional genomes included as described in methods.
Ethics oversight	Ethics oversight provided in setting of multi-institution collaborative research effort comprised of Memorial Sloan Kettering Cancer Center, New York University, Stony Brook University Hospital, Lenox Hill, Northwell Health, Columbia University, Montefiore, Cornell, and led by the New York Genome Center were included here and were previously described in (Hadi et al. 2020). Study approval was obtained via a central institutional review board (IRB), Biomedical Research Alliance of New York, and by local IRBs.

Note that full information on the approval of the study protocol must also be provided in the manuscript.

Field-specific reporting

Please select the one below that is the best fit for your research. If you are not sure, read the appropriate sections before making your selection.

☒ Life sciences ☐ Behavioural & social sciences ☐ Ecological, evolutionary & environmental sciences

For a reference copy of the document with all sections, see nature.com/documents/nr-reporting-summary-flat.pdf

Life sciences study design

All studies must disclose on these points even when the disclosure is negative.

Sample size	See Extended Data Figure 1. No sample size calculation was performed; all available genomes were used in our analysis and for each comparison sufficient numbers were determined based on an FDR-corrected p-value and magnitude of effect size.
Data exclusions	To investigate the role of complex SVs in HR-deficient cancers, we assembled a cohort of 979 predionminantly (95%) cancer WGS profiles profiles from four tumor types commonly associated with HR-deficiency (breast, ovary, prostate, and pancreas cancer; referred to as BOPP moving forward, see Methods and Supplementary Fig. 1) (Roy et al. 2011). We next sought to identify confidently BRCAld, BRCA2d, and HR-proficient cases in this BOPP cohort. We required biallelic inactivation of BRCAl or BRCA2 for a tumor to be classified as BRCAld (n=24) or BRCA2d (n=36) respectively (Riaz et al. 2017) (see Methods). We also identified 487 HR proficient BOPP samples that lacked pathogenic or rare variants in any HR-associated gene (e.g. BRCAl, BRCA2, PALB2, RADSlC; see Supplementary Table 1 for full list). We excluded the

	remaining 432 BOPP cases, which comprised tumors with monoallelic alterations and/or variants of unknown significance (VUSs) in BRCAl or BRCA2 or mutations in other HR-associated genes.
Replication	See Extended Data Figure 1. We demonstrated the robustness of SV calling by recapitulating our results with an alternative SV caller (GRIDSS) or a consensus caller, demonstrating that our results are not dependent on the choice of SV-calling algorithm.
Randomization	Not applicable as no intervention was analyzed (not possible to randomize the effect of genotype on structural variation).
Blinding	Not applicable as the outcome measured is objective (genomic structural variation), and no intervention was analyzed.

Reporting for specific materials, systems and methods

We require information from authors about some types of materials, experimental systems and methods used in many studies. Here, indicate whether each material, system or method listed is relevant to your study. If you are not sure if a list item applies to your research, read the appropriate section before selecting a response.

Materials & experimental systems

n/a	Involved in the study	
☒	☐	Antibodies
☒	☐	Eukaryotic cell lines
☒	☐	Palaeontology and archaeology
☒	☐	Animals and other organisms
☒	☐	Clinical data
☒	☐	Dual use research of concern
☒	☐	Plants

Methods

n/a	Involved in the study	
☒	☐	ChIP-seq
☒	☐	Flow cytometry
☒	☐	MRI-based neuroimaging
